# Phylogenetic and morphological analyses of *Pilosocereus leucocephalus* group *s.s.* (Cactaceae) reveal new taxonomical implications

**DOI:** 10.1007/s10265-022-01384-x

**Published:** 2022-03-19

**Authors:** Daniel Franco-Estrada, Duniel Barrios, Cristian R. Cervantes, Xochitl Granados-Aguilar, Salvador Arias

**Affiliations:** 1 Posgrado en Ciencias Biológicas, Instituto de Biología, Universidad Nacional Autónoma de México, Ciudad Universitaria, Coyoacán, 04510 Mexico City, Mexico; 2 Jardín Botánico, Instituto de Biología, Universidad Nacional Autónoma de México, Tercer Circuito Exterior, Ciudad Universitaria, Coyoacán, 04510 Mexico City, Mexico; 3 Grupo de Ecología y Conservación, Jardín Botánico Nacional, Universidad de La Habana, Carretera El Rocío km 3½, Calabazar, Boyeros, 19 230 Havana, Cuba

**Keywords:** Central America, Mesoamerican dominion, Mexico, Multivariate analysis, Phylogenetic analysis, *Pilosocereus*

## Abstract

*Pilosocereus* is one of the Cactaceae family’s most relevant genera in terms of the number of species and its wide geographical range in the Americas. Within *Pilosocereus*, five informal taxonomic groups have been recognized, one of which is *P. leucocephalus* group *s.s.*, whose phylogenetic relationships remain unresolved. Therefore, our objectives are to recognize the circumscriptions of the species in *P. leucocephalus* group *s.s.* and to corroborate the monophyly and phylogenetic relationships of this group through a set of morphological and molecular characters. This study is based on representative sampling along the broad distribution of this group in Mexico and Central America using multivariate and phylogenetic analyses. The morphological characters identified to contribute to species recognition and group formation are branch diameter, areole length, the areole length-width ratio, the distance between areoles, the length of the longest radial spine, and branch and spines colors. The chloroplast markers *rpl16*, *trnL-trnF*, and *petL-psbE* and the nuclear marker *AT1G18270* support the monophyly of the *P. leucocephalus* group *s.s.*, and two probable synapomorphies are suggested, including one transversion in *rpl16* and another in *petL-psbE*. Together, our results demonstrate that sampled species of *P. leucocephalus* group *s.s.* encompass six species distributed in Mexico and Central America: *P. alensis* and *P. purpusii* in the western region, *P. chrysacanthus* and *P. collinsii* in the central region, and *P. gaumeri* and *P. leucocephalus* in the eastern region. A taxonomic key to recognized species is provided.

**Supplementary Information:**

The online version contains supplementary material available at 10.1007/s10265-022-01384-x.

## Introduction

One of the main concerns of biology is to know the species diversity and to understand the limits between them. To know species limits is of great relevance because species represent the fundamental unit of study in multiple biological areas including ecology, population genetics, phylogenetic systematics, and botany among others (Duminil and Di Michele 2009; Su et al. 2015; Valencia-A 2020). In plants, there are many cases that use molecular and morphological evidence to know the boundaries between species complexes, for example, *Agave* L. (Asparagaceae) (Rivera-Lugo et al. 2018), *Crataegus* L. (Rosaceae) (Piedra-malagón et al. 2016), *Epidendrum* L. (Orchidaceae) (Pessoa et al. 2012), *Medicago* L. (Leguminosae) (Chen et al. 2021), *Orinus* Hitchc. (Poaceae) (Su et al. 2015), *Quercus* L. (Fagaceae) (Valencia-A 2020), and *Stenocereus* (A. Berger) Riccob. (Cactaceae) (Alvarado-Sizzo et al. 2018).

A species complex is recognized as a group of closely related species in which interspecific boundaries are unclear and are often composed of recently diverged lineages (Pinheiro et al. 2018). This occurs in the *Pilosocereus aurisetus* complex where recognition of divergent lineages using molecular markers don’t correspond precisely to the traditionally recognized species (Moraes et al. 2012). Another case, in the *Stenocereus griseus* complex, showed some genetic, ecological, and morphological differences among species (Alvarado-Sizzo et al. 2018). It most cases of species complexes, a single method may not be enough to detect the divergence of lineages. This is more difficult among recently formed species (de Queiroz 1998). Therefore, different methods and data from multiple sources increase the power to detect early stages of divergence, improving further attempts to delimitate species complexes (Leaché et al. 2009; Pinheiro et al. 2018).

In Cactaceae, there are many species complexes, probably due to variable morphological traits like the number of ribs or spines, the flower color or the size of the stems and the multiple growth forms such as the globular and globose-depressed, cylindrical, articulated or columnar (Anderson 2001; Hunt et al. 2006; Korotkova et al. 2021; Vázquez-Sánchez et al. 2012). Most of the species with a columnar growth form are members of the tribes Cereeae, Echinocereeae, and Browningieae (Anderson 2001; Hunt et al. 2006). In Cereeae, *Pilosocereus* Byles & G.D.Rowley is one of the genera with the widest distribution in the Americas compared to the remaining 13 genera of this tribe, which are restricted mostly to eastern South America (Barthlott et al. 2015; Hunt et al. 2006). Furthermore, *Pilosocereus* stands out in Cactaceae as a genus with a high number of species with 42 to 50 species (Calvente et al. 2016; Franck et al. 2019; Hunt et al. 2006).

*Pilosocereus* is defined as a tropical genus with a shrubby to treelike habit measuring up to 10 m tall, with species presenting abundant woolly flowering areoles near the apex of the branches. Although its name is derived (*pilosus* = hairy) from this latter feature, it is absent in several species, and the fruit morphology (a depressed globose, dehiscence by irregular lateral or central slits) remains the most prominent diagnostic feature (Zappi 1994). The flowers have nocturnal anthesis, nearly naked pericarpels and receptacular tubes, and few small scales and fruits with seeds measuring 1.2 to 2.5 mm long (Anderson 2001; Hunt et al. 2006; Zappi 1994). Based on a complete taxonomic revision focused on the native species of Brazil carried out by Zappi (1994), two subgenera have been recognized: *Gounellea* Zappi (two species), which was recently elevated to genus with the name *Xiquexique* Lavor, Calvente & Versieux (Lavor et al. 2020), and *Pilosocereus* (ca. 40 species), both of which are recognized based on different branching patterns (branching candelabriforms/erect or only at the base) and their fruit morphology (floral remnants not deeply embedded in the fruit apex, circular insertion points/floral remnants deeply embedded in the fruit apex, linear insertion points, respectively). The subgenus *Pilosocereus* includes five informal taxonomic groups based on habit, floral and spine morphology, and geographical distribution patterns (Hunt et al. 2006; Zappi 1994). One of these is the *P. leucocephalus* group, which broadly in *sensu lato* (*s.l.*) includes 13 species; *P. fulvilanatus*, *P. magnificus*, *P. pachycladus*, and *P. ulei* in Brazil, *P. lanuginosus*, *P. polygonus*, and *P. royenii* in the Caribbean and northern South America, and *P. alensis*, *P. chrysacanthus*, *P. collinsii*, *P. leucocephalus*, *P. purpusii*, and *P. quadricentralis* in Mexico and Central America (Table 1).

**Table 1:** Taxa associated to *Pilosocereus leucocephalus* group *s.s.*

Zappi (1994)	Hunt et al. (2006)	Calvente et al. (2016)	Lavor et al. (2018, 2020)
Extra-Brazilian species	*P. leucocephalus* group *s.l.*	*P. leucocephalus* group *s.s.*	Clade AII; Non-Brazilian species
*P. alensis*	*P. alensis*	*P. alensis*	*P. alensis*
*P. chrysacanthus*	*P. chrysacanthus *	*P. chrysacanthus *	*P. chrysacanthus*
	*P. collinsii*	*P. collinsii*	*P. collinsii*
	*P. fulvilanatus*		
*P. lanuginosus*	*P. lanuginosus*		*P. lanuginosus*
*P. leucocephalus*	*P. leucocephalus*	*P. leucocephalus*	*P. leucocephalus*
	*P. magnificus *		
	*P. pachycladus*		
*P. polygonus*	*P. polygonus*		*P. polygonus*
*P. purpusii*	*P. purpusii*	*P. purpusii*	*P. purpusii*
*P. quadricentralis*	*P. quadricentralis*	*P. quadricentralis*	*P. quadricentralis*
*P. royenii*	*P. royenii *	P. royenii*	P. royenii*
	*P. ulei*		

*In this case the correct name is *P. gaumeri* according to Franck et al. (2019)

Regarding the infrageneric classification of *Pilosocereus* (Zappi 1994), recent phylogenetic analysis did not support this classification, although a clade including *P. leucocephalus* and six other species native to Mexico and Central America was strongly supported (Calvente et al. 2016), which we refer to here as the *P. leucocephalus* group *sensu stricto* (*s.s.*) (*sensu* Calvente et al. 2016). Subsequent phylogenetic studies including all species of the *P. leucocephalus* group *s.s.* obtained similar results, but *P. lanuginosus* and *P. polygonus* were recovered as members of this group and closely related to *P. chrysacanthus* and *P. quadricentralis* (Lavor et al. 2018, 2020). Nevertheless, the phylogenetic relationships within species of this group remain unknown mainly due to the lack of a wider sampling of taxa from Mexico and Central America.

**Fig. 1:**
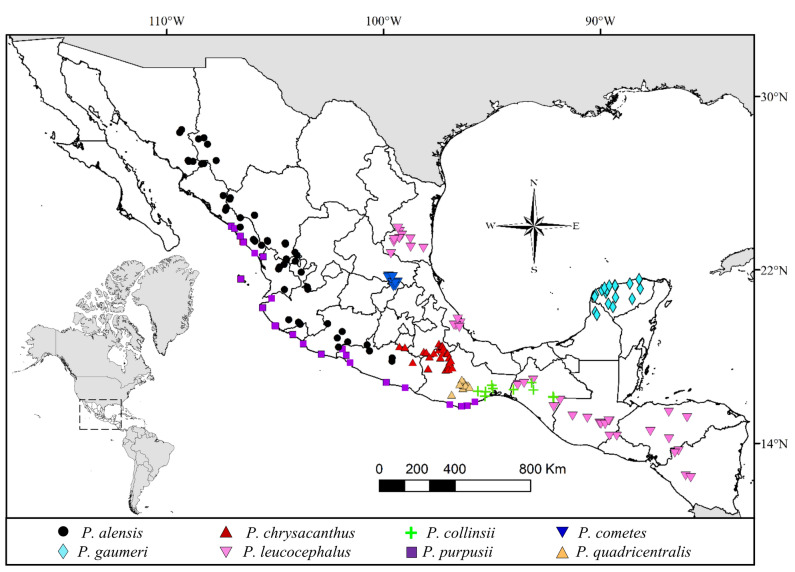
Geographical distribution of *Pilosocereus leucocephalus* group *s.s.* taxa based on georeferenced specimen records, as considered by Barthlott et al. (2015), Guzmán et al. (2003), and Yetman (2007)

Over time, there have been discrepancies in the number of recognized species inside the *P. leucocephalus* group *s.s.*, ranging from seven to eleven species (Byles and Rowley 1957; Hunt et al. 2006), with notable controversies on the recognition of *P. collinsii*, *P. cometes*, and *P. gaumeri*, suggesting *P. collinsii* as a synonym of *P. purpusii*, *P. cometes* of *P. leucocephalus*, and *P. gaumeri* of *P. royenii* (Anderson 2001; Hunt et al. 2006; Zappi 1994). As a consequence of these uncertain interspecific boundaries, there is also confusion in recognizing their geographical ranges, but the distributional pattern of this group is well known, extending from the eastern and western coasts of Mexico to Central America (Barthlott et al. 2015; Guzmán et al. 2003; Yetman 2007) (Fig. 1).

Recent studies have expanded the knowledge of *Pilosocereus* species in the Caribbean and northern South America (Calvente et al. 2016; Franck et al. 2019), however, no solid knowledge of systematics inside the *Pilosocereus leucocephalus* group *s.s.* (Calvente et al. 2016) is available. Because we only have the taxonomic contributions made by Byles and Rowley (1957) and the recent phylogenetic study by Calvente et al. (2016), which used a low number of samples within this group, the need of a broader sampling in this particular group is evident, including the use of morphological and molecular characters to better understand their phylogenetic relationships and species delimitation. Therefore, the objectives of this study are (1) to evaluate the circumscriptions of the species in the *P. leucocephalus* group *s.s.* (Calvente et al. 2016) and (2) to test monophyly and expand knowledge regarding phylogenetic relationships among this group using a set of morphological (vegetative and reproductive) and molecular characters (three plastid and one nuclear markers) as evidence.

## Materials and methods

### Taxon sampling

The focal group of this study is the recognized species of *Pilosocereus leucocephalus* group *s.s.* (Calvente et al. 2016) from Mexico and Central America—*P. alensis*, *P. chrysacanthus*, *P. collinsii*, *P. cometes*, *P. gaumeri*, *P. leucocephalus*, *P. purpusii*, and *P. quadricentralis*—based on recent taxonomic syntheses (Franck et al. 2019; Guzmán et al. 2003; Hunt et al. 2006) and a phylogenetic study of the genus *Pilosocereus* (Calvente et al. 2016). The geographical distribution of the species of *Pilosocereus leucocephalus* group *s.s.* is shown in Fig. 1. As outgroup for the phylogenetic analysis, we included some species of the Cereeae tribe and *Pilosocereus* species from South America. The functional outgroup is represented by *Browningia hertlingiana* and *Lasiocereus fulvus*, both early-divergent members in expanded Cereeae (BCT clade) (Hernández-Hernández et al. 2011; Lendel 2013).

## Data collection from herbaria and field work

**Fig. 2:**
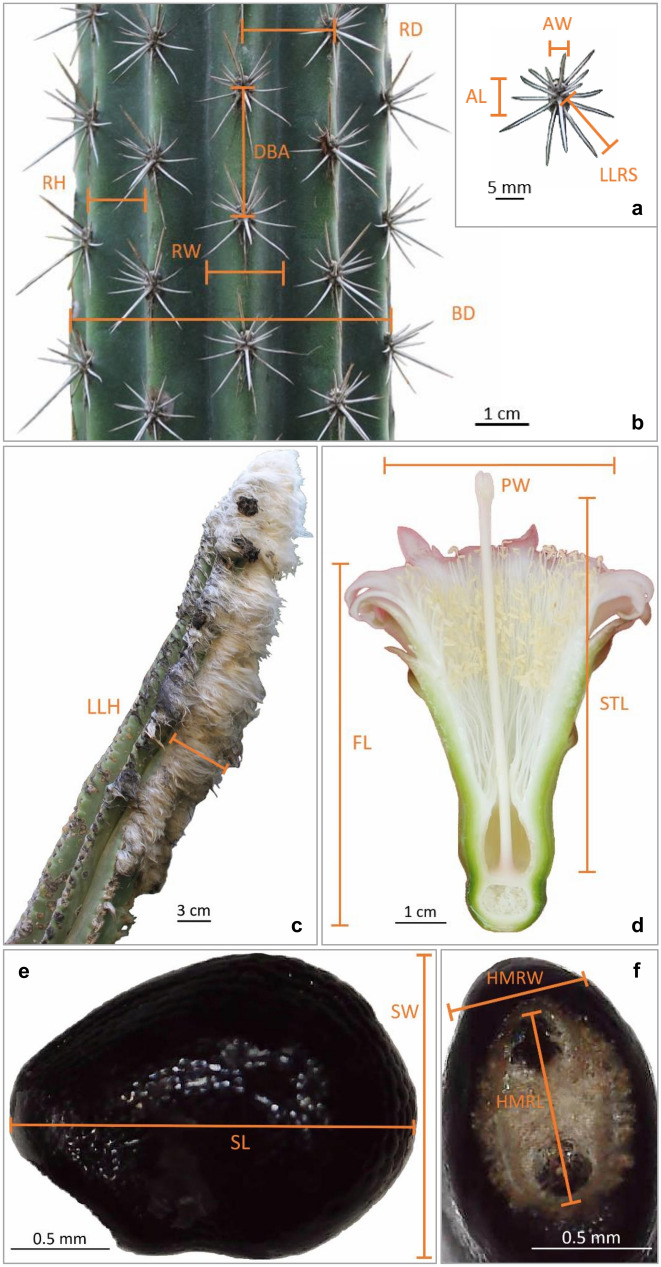
Morphological characters of *Pilosocereus leucocephalus* group *s.s.* analyzed. The abbreviations are defined in Table 2. **a**, **b)** *P. collinsii* (*DFE 67*), **c)** *P. leucocephalus* (*DFE 60*), **d)** *P. collinsii* (*DFE 40*), and **e**, **f)** *P. chrysacanthus* (*DFE 36*)

For the development of this research, 453 herbarium specimens from Instituto Politécnico Nacional, Durango (CIIDIR), Instituto Politécnico Nacional, Mexico City (ENCB), Universidad Nacional Autónoma de México, Mexico City (FCME), Herbario del Centro de Investigación en Alimentación y Desarrollo, A.C. (HCIAD), Universidad de Guadalajara (IBUG), Universidad Nacional Autónoma de México, Iztacala (IZTA), Herbario Nacional de México, Mexico City (MEXU), Instituto Politécnico Nacional, Oaxaca (OAX), Universidad Autónoma Metropolitana Iztapalapa, Mexico City (UAMIZ), Universidad Autónoma de Tamaulipas (UAT), University of Arizona (ARIZ), Harvard University (GH), The New York Botanical Garden (NY), California Botanic Garden (RSA), Universidad Nacional Autónoma de Honduras (TEFH), and Smithsonian Institution (US) were examined. Subsequently, extensive field work including all the species recognized in the *P. leucocephalus* group *s.s.* was carried out; a set of specimens was deposited in the Living Collection of the Botanical Garden, Instituto de Biología UNAM, while the voucher specimens were deposited in MEXU. At each collection site, photographs were taken with reference scales for a set of 21 morphological characters, and observations and complementary measurements were performed on the specimens deposited in the Botanical Garden (Table 2, Fig. 2). We created a database using geographic information from herbaria and our field records to construct a map of the geographical distribution of each species in ArcGIS v.10.5 (Fig. 1).

**Table 2:** Quantitative and qualitative morphological characters of *Pilosocereus leucocephalus* group *s.s.* analyzed

Characters
1. Branch diameter (BD)
2. Length of the longest radial spine (LLRS)
3. Rib height (RH)
4. Rib width (RW)
5. Rib distance (RD)
6. Areole length (AL)
7. Areole width (AW)
8. Distance between areoles (DBA)
9. Length of the longest hairs (LLH)
10. Flower length (FL)
11. Perianth width (PW)
12. Style length (STL)
13. Seed length (SL)
14. Seed width (SW)
15. Hilum-micropylar region length (HMRL)
16. Hilum-micropylar region width (HMRW)
17. Habit
18. Areole shape
19. Branch color at the apex
20. Spine color at the branch apex
21. Fertile part disposition

Quantitative characters are shown in Fig. 2 and for more details on the qualitative characters see Appendix S2

### Multivariate analysis

The evaluated morphological characters of mature plants are shown in Table 2, 16 of which were quantitative and five qualitative. The quantitative traits of branches and flowers were measured in the program ImageJ v.1.52a based on our field and greenhouse photographs. For seed measurement, a Celestron Handheld Digital Microscope with the program MicroCapture Pro v.2.4.1 was used. The arithmetic mean of branch characters was obtained by measuring three adult individuals per locality with ten measurement replicates for all samples. A total of 39 localities were sampled to measure branch characters, and for flower and seed characters, we used different sample sizes (Table S1).

Prior to our multivariate analyses, we performed the Royston test in R v.4.0.2 (R Core Team 2013) with the package MVN v.5.8 (Korkmaz et al. 2014) to evaluate whether our data had a multivariate normal distribution. Because our data did not fulfill this assumption, we transformed them into natural logarithm values to improve the distribution and homogenize their variances. Pearson correlation coefficients were calculated for the quantitative characters in R with the package corrplot v.0.84 (Wei and Simko 2017) to identify and exclude strongly correlated characters with a value greater than 0.6 (Akoglu 2018), which could lead to overestimation of the results.

Principal component analyses (PCAs) were carried out to first determine the weights of the characters contributing to the group’s formation and to show the similarities between localities and variables as points on a plane, using the function prcomp in R. The first PCA was performed using only the measurements of seven vegetative branch traits from 39 localities because the reproductive traits of flower and seed could not be obtained either in the field or in cultivation for all the localities. The second PCA was performed with 13 traits (vegetative and reproductive) from branches, flowers, and seeds for 14 localities.

Furthermore, PCAs were carried out with mixed data (numerical and categorical) to include qualitative characters with taxonomic importance, using the function PCAmix in the R package PCAmixdata v.4 allowing to perform a PCA of mixed quantitative and qualitative data (Chavent et al. 2017). The merged data table to be analyzed by PCAmixdata comprises *n* localities described by *p1* numerical variables and *p2* categorical variables. A mixed PCA was performed with the quantitative (only vegetative) and qualitative traits combined for 39 localities and another PCA mix based on the quantitative (vegetative and reproductive) and qualitative traits combined for 14 localities. The qualitative traits were habit, areole shape, colors branch at the apex and spine at the branch apex, and fertile part disposition (Table 3).

**Table 3:** Qualitative character states used in the multivariate analysis of *Pilosocereus leucocephalus* group *s.s*

Species	Habit	Areole shape	Branch color at the apex	Spine color at the branch apex	Fertile part disposition
*P. alensis*	Shrubby	Circular	Light blue green	Orange-brown	Continuous
*P. chrysacanthus*	Treelike	Circular	Light blue green	Yellow	Discontinuous
*P. collinsii*	Shrubby	Elliptic	Medium green	Dark brown	Discontinuous
*P. cometes*	Shrubby	Circular	Light blue green	Dark brown	Continuous
*P. gaumeri*	Treelike	Circular	Light green	Yellow	Discontinuous
*P. leucocephalus*	Shrubby	Circular	Light blue green	Dark brown	Continuous
*P. purpusii*	Shrubby	Circular	Medium green	Yellow	Discontinuous
*P. quadricentralis*	Treelike	Circular	Light blue green	Orange-brown	Discontinuous

Finally, linear discriminant analysis (LDA) was performed to evaluate the known group classification a priori. For LDA, the package MASS v.7.3 (Ripley et al. 2013) was used in R. Only quantitative data were used in this analysis based on seven vegetative traits from 39 localities.

### DNA extraction, amplification, and sequencing

We amplified the chloroplast markers *rpl16*, *trnL-trnF*, and *petL-psbE*, and the nuclear marker *AT1G18270* in 54 individuals of *P. leucocephalus* group *s.s.* from Mexico and Central America. For each locality, we obtained the DNA sequences for one to three individuals (Appendix S1).

Tissue samples from approximately 1-cm^3^ specimens were silica gel dried, frozen and pulverized for DNA extraction following the CTAB method (Doyle and Doyle 1987) with the modifications reported by Bustamante et al. (2016) to avoid mucilage excess. After extraction, the total genomic DNA was stored at −20 °C. PCRs were performed in volumes of 15 µL using the commercial mix “Platinum Taq” (Invitrogen). The reactions included 1.5 µL (1×) of 10× PCR buffer, 0.3 µL of BSA (0.4 %), 0.3 µL of dNTP mix, 0.2 µL of each primer (10 pmol µL^−1^), 0.5 µL of MgCl2 (1.5 µM), 0.075 µL (0.375 units) of Taq DNA polymerase, 0.5–0.7 µL template DNA and water to reach the final volume. The following primers and thermal cycle profiles were used. For the *rpl16* intron, the primers rpl161F and rpl163R (Hernández-Hernández et al. 2011) with a temperature profile of 94 °C for 5 min; 30 cycles of 94 °C for 1 min, 55 °C for 50 s, and 72 °C for 2 min; and a final extension of 72 °C for 4 min. For the *trnL-trnF* intergenic spacers, we used the primers c, d, e, and f (Taberlet et al. 1991) with a temperature profile of 94 °C for 2 min; 29 cycles of 94 °C for 30 s, 52 °C for 30 s, and 72 °C for 1 min; and a final extension of 72 °C for 7 min. For the *petL-psbE* intergenic spacer, we used the primers petL and psbE (Shaw et al. 2007) with a temperature profile of 94 °C for 2 min; 30 cycles of 94 °C for 1 min, 52 °C for 30 s, and 72 °C for 1 min; and a final extension of 72 °C for 5 min. For the *AT1G18270* intron, we used the c primers (Granados-Aguilar et al. 2021) with a temperature profile of 94 °C for 2 min; 36 cycles of 94 °C for 32 s, 56.5 °C for 30 s, and 72 °C for 1 min and 10 s; and a final extension of 72 °C for 5 min. Finally, the PCR cleaning and Sanger sequencing was performed at the Laboratorio de Biología Molecular de la Biodiversidad y de la Salud, Instituto de Biología, UNAM.

### Sequence edition and alignment

Sequences were assembled in Sequencher v.5.4. Then, matrices were generated for each marker, prealigned with Muscle v.3.8 (Edgar 2004) and manually adjusted in PhyDE v.0.9971 (Müller et al. 2005), and a 156-bp highly variable site (nonalignable) only in the nuclear region was excluded. Insertion and deletion events (indels) were identified and coded according to the simple coding method (Simmons and Ochoterena 2000).

### Phylogenetic analyses

To determine if there was incongruence between the chloroplast and nuclear data, we performed in PAUP* v.4.0 (Swofford 2003) an incongruence length difference test (ILD) (Farris et al. 1994). Partitions for each marker were designated and we performed a heuristic search with 1,000 homogeneity replicates. We organized our data into three matrices to carry out phylogenetic analyses. The first matrix includes 74 terminals for three chloroplast markers, the second matrix includes the same terminals for the chloroplast markers plus one nuclear marker, and the third includes 64 terminals for the chloroplast and nuclear markers plus morphological data of the *P. leucocephalus* group *s.s.* (Appendix S2).

Molecular phylogenies were reconstructed using probabilistic methods. For Bayesian inference analysis (BI), five partitions were required: four for DNA and one for indels. For DNA partitions, the molecular model of evolution was estimated using the Bayesian Information Criterion (BIC) as implemented in jModelTest v.2.1 (Darriba et al. 2012), and for indels partition, a restriction site model was used according to Ronquist et al. (2011). The BI analysis was performed in MrBayes v.3.2 (Ronquist et al. 2012) and consisted of two independent runs of four chains for 10,000,000 generations, sampling one tree each 1,000 generations, and starting with one random tree. In the Markov Chain Monte Carlo (MCMC) search, 25 % of the initial trees were discarded as burn-in, and with the remaining trees, a majority-rule consensus tree with nodal posterior probabilities (PP) was generated. A maximum likelihood analysis (ML) was performed in the chloroplast and chloroplast plus nuclear marker matrices in RAxML v.8.2 (Stamatakis 2014) using the default model GTR +G model and 10,000 bootstrap support (BS).

A total evidence analysis was performed under maximum parsimony (MP) and BI using molecular and morphological characters. The 16 quantitative morphological characters measured from branches were evaluated by ANOVA followed by the Tukey test to obtain feature intervals for each measured character using the package agricolae v.1.3 (de Mendiburu 2013) in R. Finally, 12 morphological characters were incorporated into the phylogenetic analysis. The MP analysis was performed in PAUP* v.4.0a168 (Swofford 2003) with a heuristic search of 1,000 random addition replicates and tree-bisection-reconnection (TBR) branch swapping and the option MulTrees, and the best score trees among 10 to 40 of the most parsimonious trees were filtered. The MaxTrees option was set at 100,000. Bootstrap analyses were performed using 1,000 replicates with TBR branch swapping and simple addition sequences. The MaxTrees option was set at 1,000 to avoid entrapment in local optima. For BI analysis, the same parameters described above were used with incorporation of the Mk model for morphological characters (Lewis 2001). Trees obtained from phylogenetic analyses were edited in FigTree v.1.4.2 (Rambaut 2014).

## Results

### Morphological analysis

The measured morphological characters are detailed in Fig. 2. The following characters were excluded due to their strong correlations with others: branch diameter, rib height, rib width, rib distance, areole length, areole width, flower length, style length, and seed width (Fig. S1). Furthermore, for subsequent analyses, we calculated the rib width-distance, areole length-width, and seed length-width ratios.

**Fig. 3:**
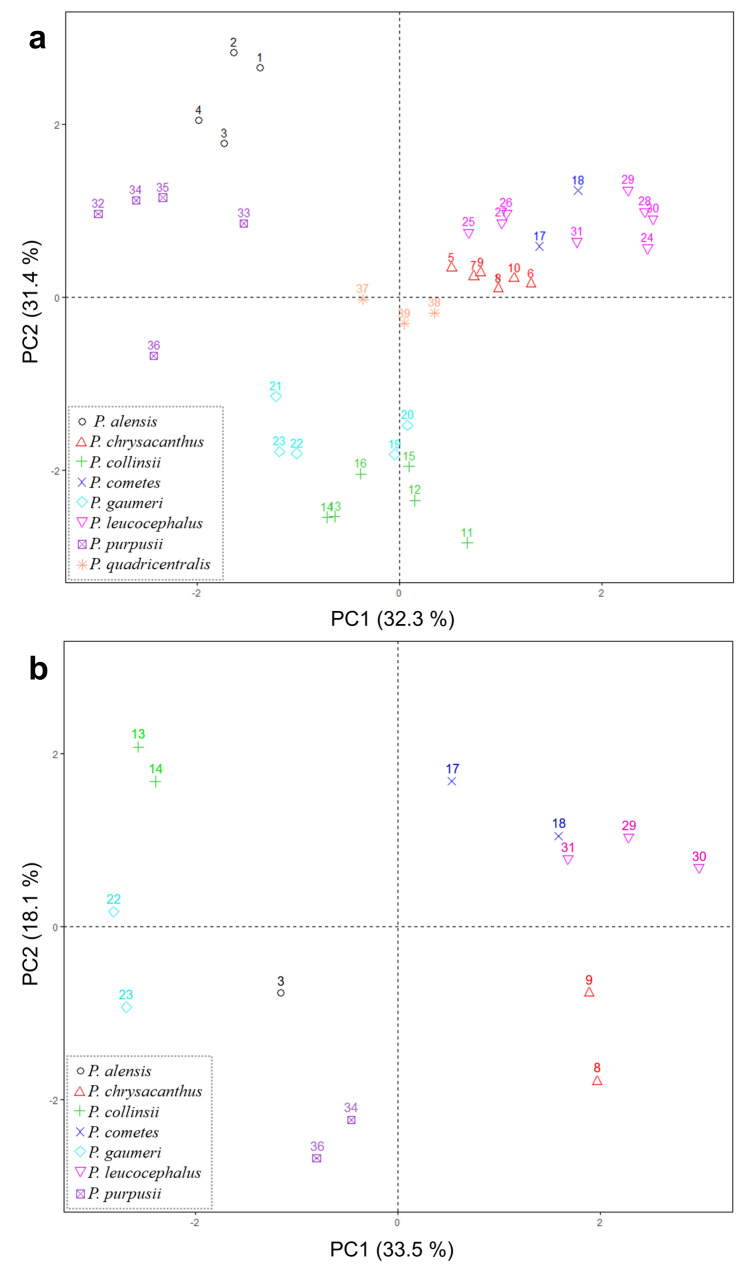
Scatter plots of the principal component analysis (PCA) of quantitative characters in *Pilosocereus leucocephalus* group *s.s.* **a)** PCA based on seven quantitative characters in specimens from 39 localities and **b)** PCA based on the vegetative and reproductive characters of 13 quantitative variables in specimens from 14 localities. Numbers refer to the ID of Table S1

We performed a PCA whereby population groups were determined using seven branch traits in 39 localities (Fig. 3a). The first two components explained 63.7 % of the variation. The traits with the highest weights in the first component were the length of the longest radial spine and areole length, while those in the second component were branch diameter and the areole length-width ratio (Table S2). In the PCA performed for 14 localities with 13 traits from branches, flowers, and seeds, we obtained 51.6 % for the explained variation, and we excluded *P. quadricentralis* due to a lack of flowers or seeds by locality (Fig. 3b).

**Fig. 4:**
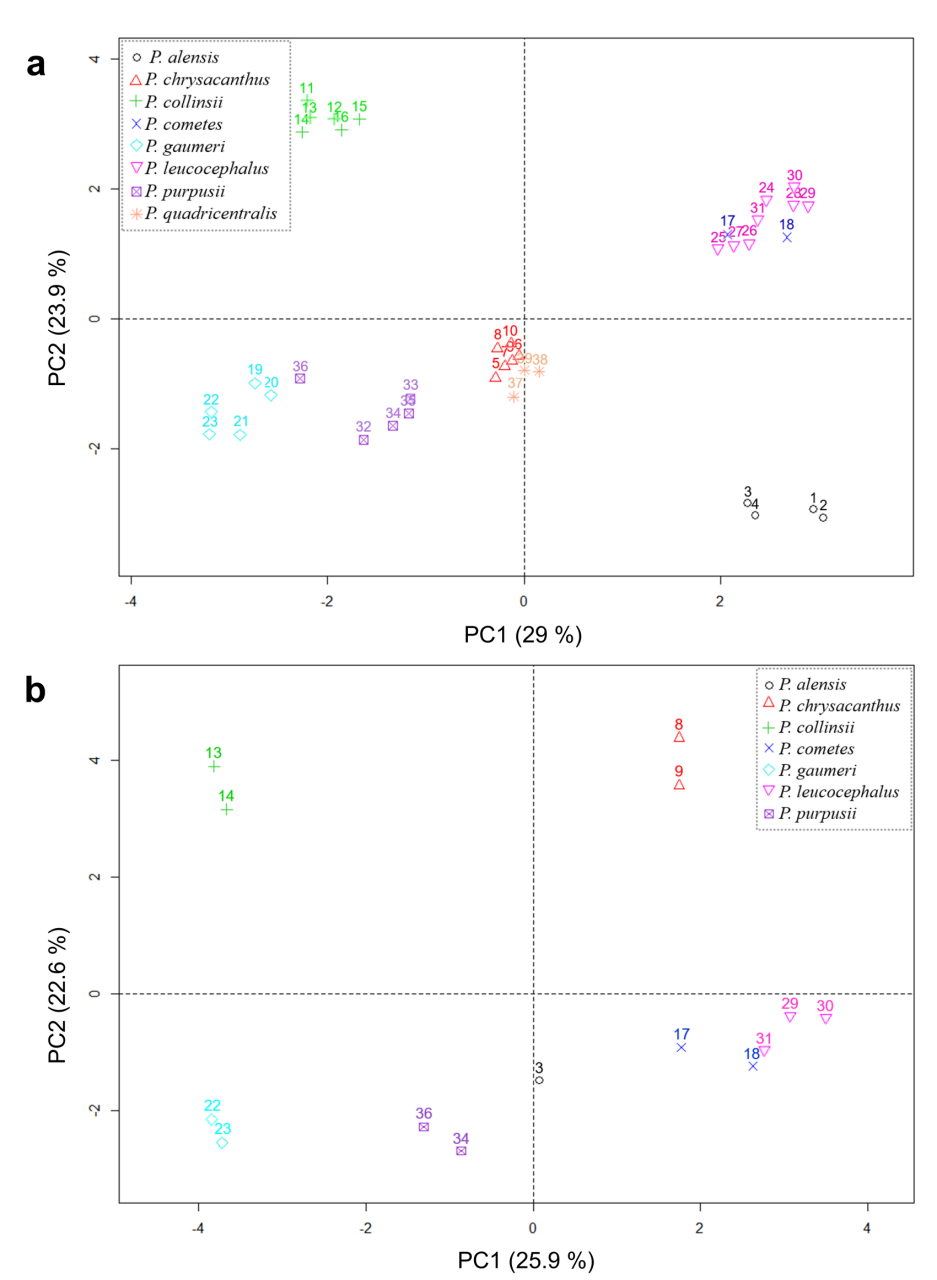
Scatter plots of the principal component analysis of mixed data (PCA mix) for quantitative and qualitative characters of *Pilosocereus leucocephalus* group *s.s.* **a)** PCA mix based on seven quantitative characters and five qualitative characters in specimens from 39 localities and **b)** PCA based on the vegetative and reproductive characters of 13 quantitative and five qualitative variables in specimens from 14 localities. Numbers refer to the ID of Table S1

The combined analysis of quantitative and qualitative traits in 39 localities (mixed PCA, Fig. 4a) revealed that the first two components explain 52.9 % of the variation, where the traits with the highest weights on the first component were the branch diameter, fertile part distribution, and branch color at the apex, while for the second component, they were the spine color at the branch apex, the distance between areoles, and areole length (Table S3). In the mixed PCA performed for 14 localities using 13 quantitative traits from branches, flowers, and seeds plus five qualitative traits, we obtained a similar result to the analysis of the 39 localities which form six species groups (Fig. 4b). The qualitative character states are shown in Table 3.

The results derived from the LDA revealed that 100 % of *P. alensis*, *P. chrysacanthus*, *P. collinsii*, *P. gaumeri*, and *P. purpusii* individuals were correctly assigned and therefore classified in their own species, while only 91.6 %, 88.8 %, and 33.3 % of *P. leucocephalus*, *P. quadricentralis*, and *P. cometes* individuals were correctly assigned, respectively (Table 4). The characters that best discriminate between the species of *P. leucocephalus* group *s.s.* are areole length-width ratio, areole length, branch diameter, and distance between areoles (Table S4).

**Table 4:** Evaluation of taxonomic identification errors under linear discriminant analysis based on seven quantitative characters of *Pilosocereus leucocephalus* group *s.s.* from 39 localities

	*P*. *alensis*	*P. chrysacanthus*	*P. collinsii*	*P. cometes*	*P. gaumeri*	*P. leucocephalus*	*P. purpusii*	*P. quadricentralis*	Total
*Pilosocereus alensis*	12	0	0	0	0	0	0	0	12/12
*Pilosocereus chrysacanthus*	0	18	0	1	0	1	0	1	18/18
*Pilosocereus collinsii*	0	0	18	0	0	0	0	0	18/18
*Pilosocereus cometes*	0	0	0	2	0	1	0	0	2/6
*Pilosocereus gaumeri*	0	0	0	0	15	0	0	0	15/15
*Pilosocereus leucocephalus*	0	0	0	3	0	22	0	0	22/24
*Pilosocereus purpusii*	0	0	0	0	0	0	15	0	15/15
*Pilosocereus quadricentralis*	0	0	0	0	0	0	0	8	8/9
Percentage	100	100	100	33.3	100	91.6	100	88.8	

### Phylogenetic analyses

Phylogenetic analyses included 74 terminals with 263 sequences, 237 of which were new sequences, while 26 were sequences from GenBank, mainly from the previous work of Calvente et al. (2016) and Schlumpberger and Renner (2012) (Appendix S1). According to the ILD test, no significant incongruence was found between the plastid and nuclear data (*P* = 0.49), thus all markers were concatenated and analyzed combined. The concatenate matrix with all markers consisted of 3,265 nucleotides and 19 indels, 83 of which were informative (2.54 %), with a greater number of informative characters in *rpl16* (27). Finally, the 19 indels mainly included insertions and deletions with only one inversion. Data of molecular evolution models for each marker and the concatenate matrix as well as the number of indels are shown in Table S5.

**Fig. 5:**
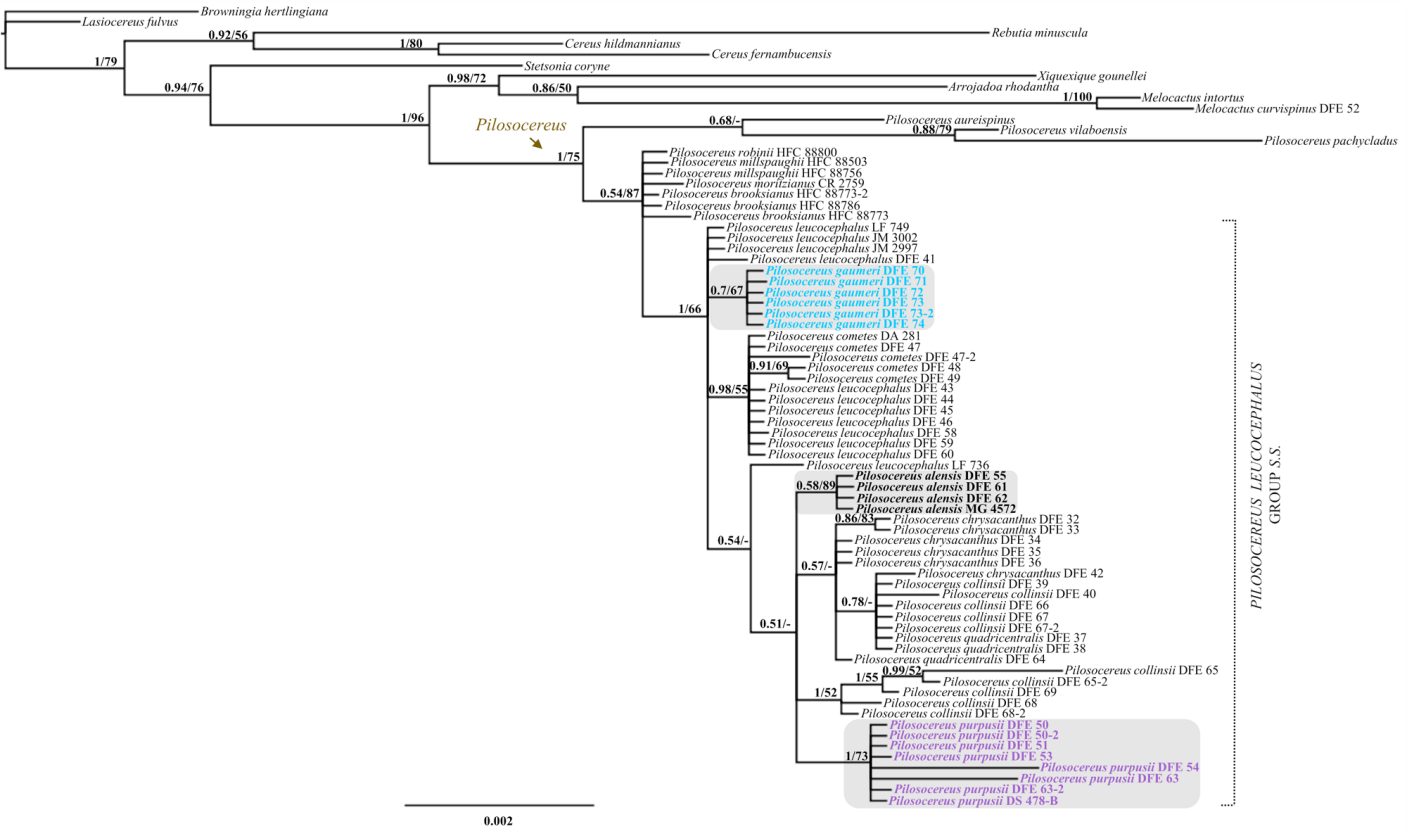
Majority-rule consensus tree of 15,002 trees resulting from the Bayesian analysis based on the concatenation of three chloroplast DNA markers (*rpl16*, *trnL-trnF*, *petL-psbE*) and indels. The value at the node corresponds to PP/BS from the BI/ML analyses. The dotted line delimits *Pilosocereus leucocephalus* group *s.s.*, and within the group, the names of the species that recover under the criterion of reciprocal monophyly are highlighted

**Fig. 6:**
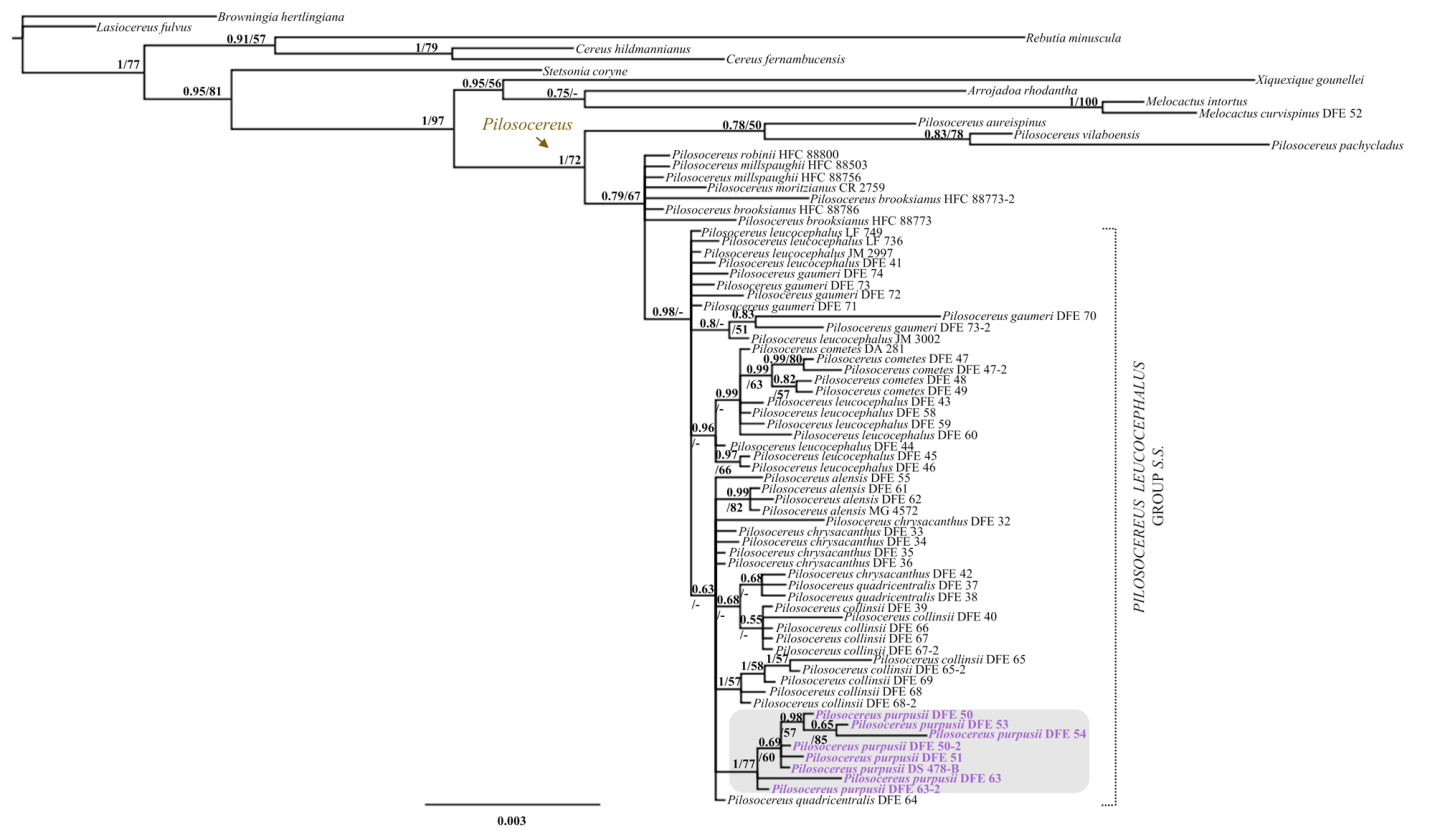
Majority-rule consensus tree of 15,002 trees resulting from the Bayesian analysis based on the concatenation of three chloroplast DNA markers (*rpl16*, *trnL-trnF*, *petL-psbE*), indels, and one nuclear marker (*AT1G18270*). The value at the node corresponds to PP/BS from the BI/ML analyses. The dotted line delimits *Pilosocereus leucocephalus* group *s.s.*, and within the group, the name of the species that recovers under the criterion of reciprocal monophyly is highlighted

Our results in the analysis performed under BI and ML were highly congruent, the Bayesian majority-rule consensus tree in Fig. 5 has only plastid markers whereas the tree in Fig. 6 also includes a nuclear gene. The genus *Pilosocereus* is recovered as monophyletic with high to moderate support (Fig. 5; 1 PP/75 % BS). Within *Pilosocereus*, two main clades can be recognized—one with the species included from Brazil: *P. aureispinus*, *P. pachycladus*, and *P. vilaboensis* (Fig. 5; 0.68 PP/− BS); and the second with all species from Mexico and Central America highlighted as *P. leucocephalus* group *s.s.*, which are closely related to *P. brooksianus*, *P. millspaughii*, and *P. robinii* from the Caribbean and to *P. moritzianus* from northern South America (Fig. 5; 0.54 PP/87 % BS). *P. leucocephalus* group *s.s.* is monophyletic with high to low support (Fig. 5; 1 PP/66 % BS). This clade includes 54 terminals and within this were recovered at species level three clades: *P. alensis* (Fig. 5; 0.58 PP/89 % BS), *P. gaumeri* (Fig. 5; 0.7 PP/67 % BS), and *P. purpusii* (Fig. 5; 1 PP/73 % BS).

The majority-rule consensus tree for three plastid markers and one nuclear marker showed similar results to those obtained with only plastid markers. *P. leucocephalus* group *s.s.* is monophyletic with high support in BI (Fig. 6; 0.98 PP/− BS), and within this clade, only *P. purpusii* is recovered as monophyletic with high to moderate support (Fig. 6; 1 PP/77 % BS). In the analyses performed with only plastid markers and plastid plus nuclear markers, within the *P. leucocephalus* group *s.s.*, *P. chrysacanthus*, *P. collinsii*, *P. cometes*, *P. leucocephalus*, and *P. quadricentralis* were not recovered as monophyletic.

**Fig. 7:**
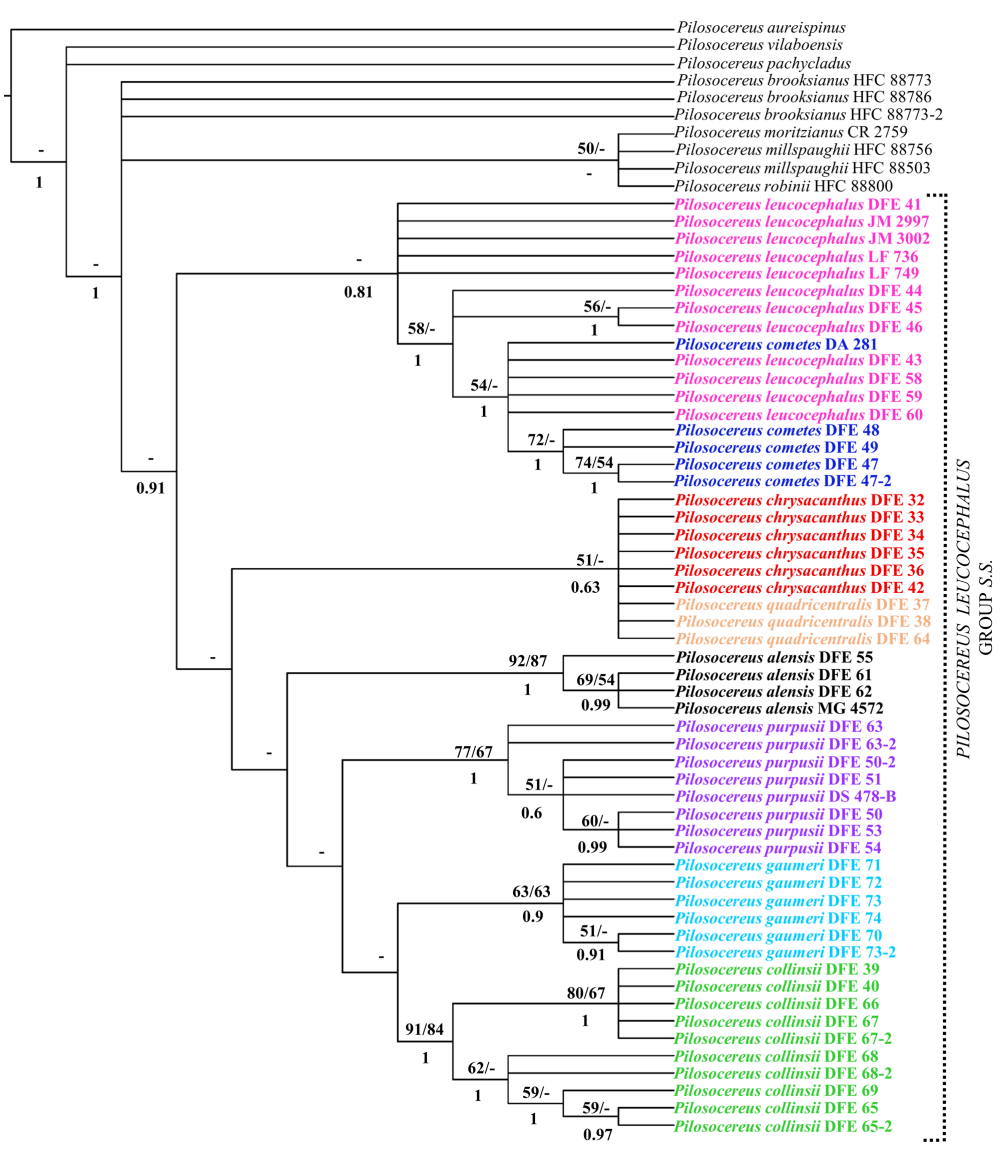
Strict consensus tree of the 31 most parsimonious trees resulting from the MP analysis based on molecular (*rpl16*, *trnL-trnF*, *petL-psbE*, *AT1G18270*, and indels) and morphological data (five quantitative and seven qualitative characters). The value at the top branches corresponds to BS/JK from the MP analysis, at the bottom branches corresponds to PP from the IB analysis, and a dash indicates a lack of support. The dotted line delimits *Pilosocereus leucocephalus* group *s.s.*, and the colors of the terminals denote the taxa according to Guzmán et al. (2003)

For the total evidence analysis using molecular and morphological characters, *P. leucocephalus* group *s.s.* was recovered with high support only in the Bayesian analysis (Fig. 7; 0.91 PP). Within this group, six clades at the species level were recovered with *P. cometes* nested in *P. leucocephalus* as well as *P. chrysacanthus* and *P. quadricentralis* in the same clade. The first clade was integrated by all terminals for *P. leucocephalus* and *P. cometes* (Fig. 7; 0.81 PP) and is sister to the remaining clades. The *P. chrysacanthus*-*P. quadricentralis* clade (Fig. 7; 51 % BS/0.63 PP) is sister to the clades *P. alensis* (Fig. 7; 92 % BS/87 % JK/1 PP), *P. collinsii* (Fig. 7; 91 % BS/84 % JK/1 PP), *P. gaumeri* (Fig. 7; 63 % BS/63 % JK/0.9 PP), and *P. purpusii* (Fig. 7; 77 % BS/67 % JK/1 PP), which were recovered as monophyletic. The relationships between species do not have support. Last, for the *P. leucocephalus* group *s.s.*, two putative synapomorphies can be recognized, including a transversion in *rpl16* (T → A) and another in *petL-psbE* (C → G). At a more inclusive level, we recognized only autapomorphies in four species: for *P. alensis*, the long hairs (7–12 cm) and a deletion of 22 bp in *trnL-trnF*; for *P. purpusii*, an insertion of 4 bp in *trnL-trnF* and a transversion in *petL-psbE* (C → A); for *P. gaumeri*, an insertion of 7 bp in *rpl16*; and in *P. collinsii*, the areole length-width ratio and elliptical areoles.

## Discussion

### Recognition of species with morphological attributes

**Fig. 8:**
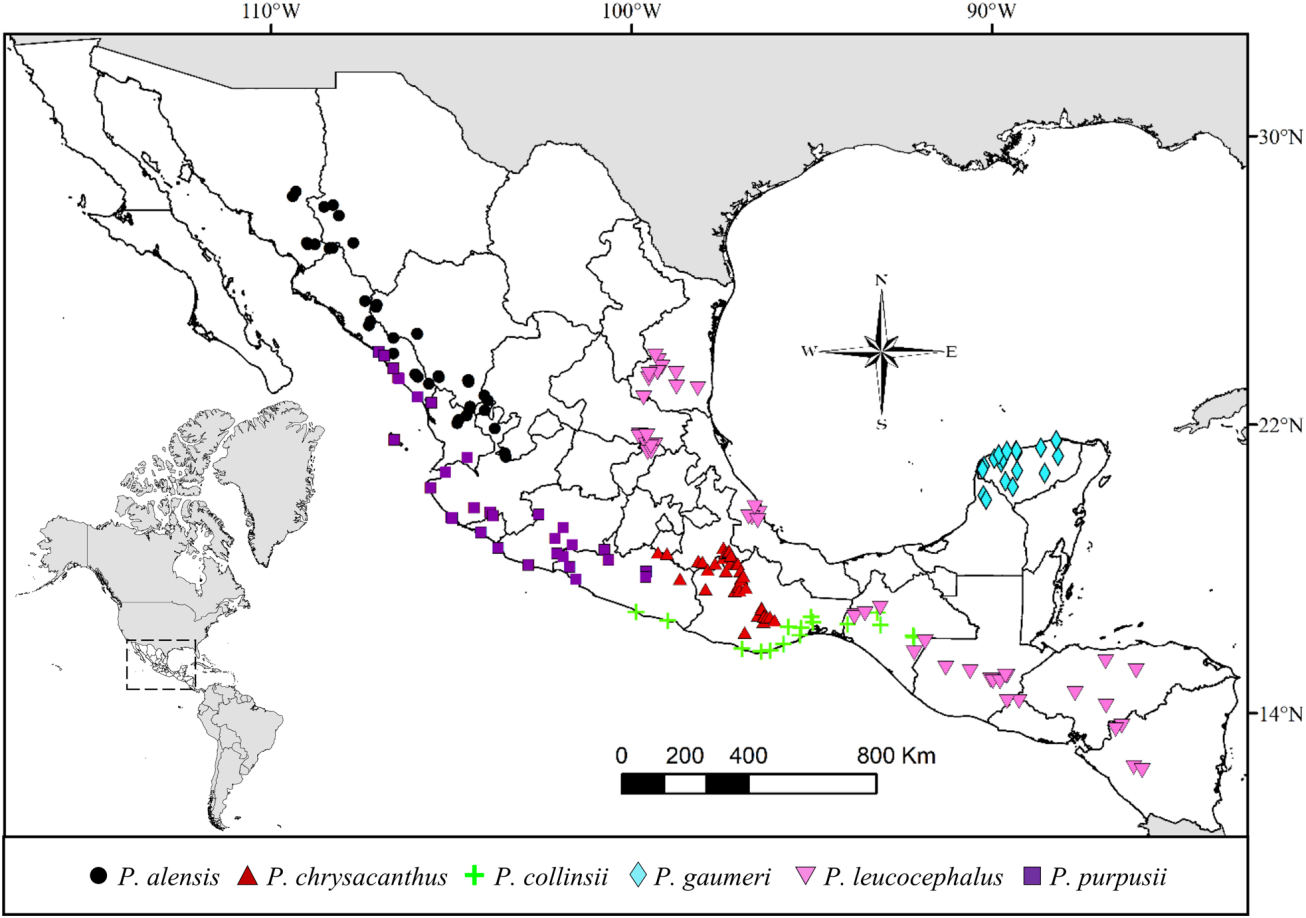
Geographical distribution of *Pilosocereus leucocephalus* group *s.s.* taxa based on georeferenced specimen records, as considered in this work

Based on our results, six species are recognized as having the following combinations of characters. 1) *P. alensis* is characterized by shrubby habit with small areoles (2–3 mm), orange-brown spines, long hairs (7–15 cm), and large seeds (approximately 2.5 mm long). It is distributed in western Mexico, from Sonora to Jalisco. 2) *P. chrysacanthus* comprises treelike habit with branches measuring 7.5–12 cm in diameter, usually yellow spines, short hairs (2.5–5 cm) and flowers measuring 6 to 10 cm long. It is distributed in southern Mexico in Guerrero, Morelos, Oaxaca, and Puebla. 3) *P. collinsii* includes shrubby habit with elliptical areoles, a distance between areoles of 1.7–2.2 cm and dark brown spines. It is distributed in southern Mexico in Chiapas, Guerrero, and Oaxaca. 4) *P. gaumeri* presents treelike habit with branches measuring 4.1–5.2 cm in diameter, light green branches, and noticeably short hairs (1.7–2.3 cm). It is distributed in southeastern Mexico, in Campeche and Yucatán. 5) *P. leucocephalus* presents shrubby and treelike habit, usually shrubs, with branches measuring 9 to 14 cm in diameter, high ribs (1.8–3.5 cm), and hairs of 4 to 8 cm in length. It is distributed from eastern Mexico (Chiapas, Querétaro, San Luis Potosí, Tamaulipas, and Veracruz) to Central America (El Salvador, Guatemala, Honduras, and Nicaragua). 6) *P. purpusii* is characterized by shrubby habit with branches measuring 6 to 9 cm in diameter, low ribs (7–16 mm), areole lengths of approximately 3 mm, and yellow spines. It is distributed in western Mexico, from Sinaloa to Guerrero (see the geographical distribution of all taxa in Fig. 8). For further information, see the taxonomic key after the taxonomic treatment.

For species recognition, *P. collinsii* has been considered synonymous with *P. purpusii* because they share some characters, including a shrubby habit and similar branch diameters, radial spine lengths and rib heights (Anderson 2001; Yetman 2007; Zappi 1994). Our results confirm that they are different species. Based on the PCA, the characters with the highest weights (loadings) contributing to different groups are those associated with the areole, distance between areoles, and colors of spines (Tables 3, 4), and in the LDA, the 18 sampled *P. collinsii* individuals were all correctly classified in this species. Similarly, for *P. purpusii*, the 15 sampled individuals were all correctly classified (Table 4). *P. gaumeri* had previously been designated as synonym for *P. royenii* because they share the treelike habit, yellow spines, and flowers of approximately 7 cm long (Anderson 2001; Barthlott et al. 2015; Hunt et al. 2006; Zappi 1994). However, according to Franck et al. (2019), they are different species considering branching (divergent/ascending to strict), stem thickness (slender/thick), and fruit color (purple/red). Our morphological measurements in *P. gaumeri* which agree with the diagnostic characters presented by Franck et al. (2019), including the hair length of 1.7 to 2.3 cm for *P. gaumeri* while *P. royenii* has hairs up to 4 cm long. Therefore, these remarkable morphological differences between *P. gaumeri* and *P. royenii* allows us to support them as different species.

On the other hand, previous knowledge of *P. cometes* and *P. leucocephalus* shows that the main difference between them is the length of hairs, which is short (2 cm) in *P. cometes* and long (10 cm) in *P. leucocephalus*, thus considering them to be independent species (Bravo-Hollis 1978; Britton and Rose 1920; Byles and Rowley 1957; Guzmán et al. 2003). However, in this study, we found hair lengths from 4 to 8 cm for both taxa, and the LDA results show that only 2 of 6 *P. cometes* individuals sampled were correctly classified in this species, including an *P. chrysacanthus* individual and three *P. leucocephalus* individuals. Conversely, 22 of 24 sampled *P. leucocephalus* individuals were correctly classified in this species, including one *P. chrysacanthus* individual and one *P. cometes* individual (Table 4). These results support the previous proposal to consider *P. cometes* as a synonym of *P. leucocephalus* (Anderson 2001; Hunt et al. 2006; Zappi 1994). For *P. chrysacanthus* and *P. quadricentralis*, the main differences were yellow spines in *P. chrysacanthus* and orange-brown spines in *P. quadricentralis*, without recognition of central spines in *P. chrysacanthus* and up to four spines in *P. quadricentralis*, and the distances between areoles were approximately 1 cm in *P. chrysacanthus* and usually 1.5–1.7 cm in *P. quadricentralis* (Anderson 2001; Bravo-Hollis 1978; Hunt et al. 2006). Nevertheless, based on the results of mixed PCA, no clear distinction was evident between *P. chrysacanthus* and *P. quadricentralis* (Fig. 4a), and the LDA showed that 1 of 9 *P. quadricentralis* individuals was classified as *P. chrysacanthus*, while all the sampled (18) *P. chrysacanthus* individuals were correctly classified in this species (Table 4). Therefore, here, these species are considered a unique entity with shades of yellow to orange-brown coloration in spines, 1–4 central spines on the middle part of mature branches, and a distance between areoles of 1.2 to 2.2 cm, in addition to an areole size of 3.5 to 5.5 mm length and 3 to 5 mm width.

This study reveals that the morphological variation among the species of *Pilosocereus* distributed in Mexico and Central America is very narrow or has overlapping ranges; for example, a diameter of branches between 7.5 and 14.5 cm, a length of longer radial spines between 1 and 2 cm, an areole length from 3.5 to 5.3 mm, and a distance between areoles from 1.3 to 2.3 cm overlap between *P. chrysacanthus* (= *P. quadricentralis*) and *P. leucocephalus* (= *P. cometes*). Nevertheless, the contributions of five qualitative characters with taxonomic importance allowed us to maximize the differences between groups and showed a clear distinction between *P. collinsii* and *P. purpusii* (Fig. 4a). In some groups of Cactaceae, several morphological characters with continuous variation have been recognized between related species, but only some characters offer information to recognize their circumscription at the species level, as in, for example, *Cylindropuntia* (*C. multigeniculata* and *C. whipplei*: Baker 2016), *Echinocereus* (*E. acanthosetus* and *E. pulchellus*: Sánchez et al. 2020), and *Escobaria* (*E. guadalupensis* and *E. sneedii*: Baker and Johnson 2000). Similarly, in the study group, a gradation can be observed for branch diameter and the distance between areoles, but notably, *P. leucocephalus* group *s.s.* species have areas of distribution that do not overlap, namely, they are almost completely allopatric (Fig. 8). Regarding reproductive structures, the seed is revealed as potentially informative because the length of the hilum-micropylar region is among the weightiest characters in the PCA for species group formation. Future studies on seed morphology may provide more information on its taxonomic value, as has been recognized for other groups of cacti (e.g., *Melocactus*: Lemus-Barrios et al. 2021; *Stenocereus*: Arroyo-Cosultchi et al. 2006).

### Monophyly in the *P. leucocephalus* group *s.s.*

Sampled members of *P. leucocephalus* group *s.s.* were recovered in a monophyletic clade with high to low support (Figs. 5–7), with 54 terminals incorporated into the analysis and six clades at the species level distributed in Mexico and Central America (Fig. 8). According to this result, the monophyly of *P. leucocephalus* group *s.s.* reported by Calvente et al. (2016) is corroborated. For this group, two base pairs were recognized as putative synapomorphies, including one in *rpl16* (an A in site 729 from the alignment) and one in *petL-psbE* (a G in site 277 from the alignment), and a combination of morphological characters indicated the taxonomic treatment section.

Based on our results and following the regionalization of the neotropical region (Morrone 2014), the members of *P. leucocephalus* group *s.s.* included in this study are distributed in the Mesoamerican dominion and are suggested to belong to a Mesoamerican clade that is sister to the species from the Caribbean and northern South America included in our analyses, including *P. brooksianus*, *P. millspaughii*, *P. moritzianus*, and *P. robinii sensu* Franck et al. (2019). Previous phylogenetic analyses focused on *Pilosocereus* mainly from South America (Calvente et al. 2016), reported that *P. leucocephalus* group *s.s.* was integrated by *P. alensis*, *P. purpusii*, *P. leucocephalus*, *P. collinsii*, *P. chrysacanthus*, *P. quadricentralis*, and *P. gaumeri* (as *P. royenii*: mistaken name; for more details, see Franck et al. (2019)). In subsequent analyses (Lavor et al. 2020) including more species, a clade integrated by *P. alensis*, *P. chrysacanthus*, *P. collinsii*, *P. gaumeri*, *P. lanuginosus*, *P. leucocephalus*, *P. polygonus*, *P. purpusii*, and *P. quadricentralis* was recovered, which was named a non-Brazilian species clade, and the former name—*P. leucocephalus* group *s.s.*—was omitted. Thus, although our results are in agreement with those of Calvente et al. (2016), the inclusion of a higher number of Caribbean and northern South American species can help to define whether *P. leucocephalus* group *s.s.* recovers as a natural group or is only an artifact of an artificial classification, as observed for the rest of the informal *Pilosocereus* groups (Calvente et al. 2016). Furthermore, wider sampling of non-Brazilian *Pilosocereus* species may help to clarify whether the Mesoamerican clade and the species from the Caribbean and northern South America are sister groups or whether the Mesoamerican clade is nested within the clade AII or non-Brazilian clade (Lavor et al. 2018, 2020).

Interestingly, similar results to those obtained for *P. leucocephalus* group *s.s.* have been recorded in other groups of organisms with wide geographical distribution in the Americas, recognizing monophyletic groups that are distributed in the Mesoamerican dominion. For instance, among plants, Granados et al. (2017) found that in Poales, the genus *Tillandsia* L. subg. *Tillandsia* (which belongs to the clade K in the study with 82 spp.) shows a similar pattern in North and Central America. The same pattern is observed in the genus *Zamia* L. (Cycadales) (Calonje et al. 2019) with a group of 18 species restricted to the Mesoamerican dominion. While in animals, inside the genus *Sturnira* Gray (Chiroptera) the *S. parvidens* lineage from tropical areas of Mexico to northern Costa Rica is closely related to their South American sisters (Hernández-Canchola and León-Paniagua 2017). Similarly, in the genus *Alouatta* Lacépède (Primates) a Mesoamerican clade is strongly supported, comprising *A. paliatta* and *A. pigra* (Doyle et al. 2021).

### Internal relationships in *P. leucocephalus* group *s.s.*

The monophyly of *P. leucocephalus* group *s.s.* is a corroborated hypothesis, but the interrelationships of its species remain quite ambiguous. For example, in the work of Calvente et al. (2016), the authors report *P. gaumeri* as a sister to the remaining species, whereas in Lavor et al. (2018), *P. gaumeri* and *P. collinsii* are sisters of two clades, one supporting the relationships of *P. leucocephalus* with *P. purpusii* and *P. alensis* and other with *P. chrysacanthus* (= *P. quadricentralis*). In both works, *P. gaumeri* was a basal terminal, and a close relationship was identified between *P. leucocephalus*, *P. purpusii*, and *P. alensis*. A more recent analysis showed basal polytomy in *P. leucocephalus* group *s.s.* (non-Brazilian species) and maintenance of the aforementioned relationships, although an additional terminal of *P. leucocephalus* is a sister to *P. gaumeri* (Lavor et al. 2020). In our study, we were not able to recover the relationships within the group using only molecular markers, but notably, the molecular data are consistent with the morphological recognition of *P. alensis*, *P. gaumeri*, and *P. purpusii*. The problem of the poor resolution of phylogenetic relationships between species is probably associated with the recent divergence of *P. leucocephalus* group *s.s.* (a mean divergence of 0.90 million years with an interval of 1.77–0.31; Lavor et al. 2018), incomplete lineage sorting and/or long generational times. Similar situations occur in other plant groups, such as *Myosotis* (Meudt et al. 2015), *Astragalus* (Bagheri et al. 2017), and *Agave* (Jiménez-Barron et al. 2020), which also show recent diversification, which complicates determination of the phylogenetic relationships between their species.

On the other hand, when jointly analyzing morphological and molecular characters, we found clear recognition of six species and their probable internal relationships within *P. leucocephalus* group *s.s.* This analysis allows us to infer the lineages *P. alensis*, *P. chrysacanthus* (including *P. quadricentralis*), *P. collinsii*, *P. gaumeri*, *P. leucocephalus* (including *P. cometes*), and *P. purpusii* at the species level. Within *P. leucocephalus* group *s.s.*, the relationships lack support and differ from the results obtained in previous works (Calvente et al. 2016; Lavor et al. 2018, 2020), mainly because we identified *P. leucocephalus* as the sister species to the remaining species constituting the group, and *P. collinsii* appears to be the sister species of *P. gaumeri*.

In conclusion, the group *Pilosocereus leucocephalus s.s.* distributed in Mexico and Central America is supported as a monophyletic group, in which we recognize six species based on morphological and molecular characters. The most important morphological characters that contribute to the formation of groups and in being able to correctly discriminate between certain species are areole length, branch diameter, distance between areoles, and spines colors, for which its potential use in other *Pilosocereus* species is suggested. Regarding molecular characters, only *P. alensis*, *P. gaumeri*, and *P. purpusii* were recovered with reciprocal monophyly using chloroplast markers, although by including a nuclear marker only *P. purpusii* was recovered. Given the uncertainty in the taxonomic circumscription of the closely related species in this group with the previous suggestion of a likely recent divergence, the combination of morphological and molecular characters offers good results in the delimitation of its species and reveals as one same species *P. chrysacanthus* and *P. quadricentralis* as well as *P. cometes* and *P. leucocephalus*, while *P. collinsii* and *P. purpusii* turned out to be distinct species, and *P. gaumeri* closely related to the Mesoamerican species, differing from the Caribbean. For future research, we suggest that other unexplored characters should be evaluated, such as chromosome numbers or anatomical information, and conduct genomic or microsatellite analyses to broaden our knowledge of this rather complex group.

### Taxonomic treatment

The *P. leucocephalus* group *s.s.* occurring in Mexico and Central America is characterized here with amendment of the proposal made by Hunt et al. (2006) as follows: shrubby cacti, very rarely treelike, between 3.5 and 10 m high; thick branches, not very woody except at the base, often glaucous, usually 7 to 15 ribs; few to many spines, mostly thick, differentiated into central and radial; weakly differentiated floriferous areoles with long dense tufts of hairs; medium to large flowers with straight tubes, medium to large seeds (rarely small), and smooth and shiny cuticles.

Based on the results of this study, we recognize the following six species in the *P. leucocephalus* group *s.s.*:

1. ***Pilosocereus alensis*** (F.A.C.Weber) Byles & G.D.Rowley, Cact. Succ. J. Gr. Brit. 19: 66. 1957. ≡ *Pilocereus alensis* F.A.C.Weber ex Rol.-Goss., Bull. Mus. Hist. Nat. (Paris) 11(6): 508 (−509). 1905. ≡ *Cephalocereus alensis* (F.A.C.Weber) Britton & Rose, Contr. U.S. Natl. Herb. 12: 415. 1909. ≡ *Cereus alensis* Vaupel, Monatsschr. Kakteenk. 23: 23, 24, 83. 1913. TYPE: Mexico, Jalisco, Sierra del Alo, near Manzanillo, *L.Diguet s.n.* (holotype: P?; isotypes: US?, RB 00537920!).
2. ***Pilosocereus chrysacanthus*** (F.A.C.Weber ex K.Schum.) Byles & G.D.Rowley, Cact. Succ. J. Gr. Brit. 19: 66. 1957. ≡ *Pilocereus chrysacanthus* F.A.C.Weber ex K.Schum., Gesamtbeschr. Kakt. 178. 1897. ≡ *Cereus chrysacanthus* Orcutt, W. Amer. Sci. 13: 63. 1902. ≡ *Cephalocereus chrysacanthus* Britton & Rose, Contr. U.S. Natl. Herb. 12: 416. 1909. ≡ *Cephalophorus chrysacanthus* (F.A.C.Weber) Boom, Succulenta (Netherlands) 46: 107. 1967. TYPE: Mexico, near Tehuacán, *Weber s.n.* (not preserved). NEOTYPE (designed by Zappi, Succ. Pl. Res. 3: 144. 1994): Mexico, Puebla, Tehuacán, 30 Aug to 08 Sep 1905, *J.N.Rose, J.H.Painter & J.S.Rose 9993* (neotype: US 00170926!; isoneotype: NY 02256593!).
   = *Pilocereus tehuacanus* Weing., Z. Sukkulentenk. 3: 58. 1927. ≡ *Cephalocereus tehuacanus* (Weing.) Borg, Cacti (Borg), ed. 2. 150. 1951. ≡ *Pilosocereus tehuacanus* (Weing.) Byles & G.D.Rowley, Cact. Succ. J. Gr. Brit. 19: 69. 1957. TYPE: Mexico, Puebla, Tehuacán area, *C.A.Purpus s.n.*, 1907 (not preserved).
   = *Cephalocereus quadricentralis* E.Y.Dawson, Allan Hancock Found. Publ. Occas. Pap. 1: 14, tab. 3, fig. 5. 1948. ≡ *Pilosocereus quadricentralis* (E.Y.Dawson) Backeb., Cactaceae (Backeberg) 4: 2437. 1960. TYPE: Mexico, Oaxaca, east of Oaxaca-Chiapas, Pan-Pacific Highway, 1,000 m, 25 Jan 1947, *E.Y.Dawson 3004* (holotype: AHH 8259). Note: holotype transferred to RSA 0008868!. **Synon. nov.**
3. ***Pilosocereus collinsii*** (Britton & Rose) Byles & G.D.Rowley, Cact. Succ. J. Gr. Brit. 19: 66. 1957. ≡ *Cephalocereus collinsii* Britton & Rose, Cactaceae (Britton & Rose) 4: 269, fig. 242. 1923. ≡ *Pilocereus collinsii* (Britton & Rose) F.M.Knuth, Kaktus-ABC [Backeb. & Knuth] 330. 1936. LECTOTYPE (designed by Zappi, Succ. Pl. Res. 3: 150. 1994): Mexico, Oaxaca, Tehuantepec [*O.F.Cook* & *G.N.Collins s.n.*, 1902] (lectotype: US 00115537!; isolectotypes: NY 00118700!, 00120552!).
4. ***Pilosocereus gaumeri*** (Britton & Rose) Backeb., Cactaceae (Backeberg) 4: 2462. 1960. ≡ *Cephalocereus gaumeri* Britton & Rose, Cactaceae (Britton & Rose) 2: 47. 1920. ≡ *Cereus gaumeri* Standl., Publ. Field Mus. Nat. Hist., Bot. Ser. 3: 366. 1930. ≡ *Pilocereus gaumeri* (Britton & Rose) F.M.Knuth, Kaktus-ABC [Backeb. & Knuth] 330. 1936. LECTOTYPE (designed by Zappi, Succ. Pl. Res. 3: 151. 1994): Mexico, Yucatán, Progreso, 1918, *G.F.Gaumer 23934* (lectotype: US 00115539!; isolectotypes: NY 00120553!, 00120554!, 00120555!).
5. ***Pilosocereus leucocephalus*** (Poselg.) Byles & G.D.Rowley, Cact. Succ. J. Gr. Brit. 19: 67. 1957. ≡ *Pilocereus leucocephalus* Poselg., Allg. Gartenzeitung (Otto & Dietrich) 21: 126. 1853. ≡ *Cephalocereus leucocephalus* Britton & Rose, Contr. U.S. Natl. Herb. 12: 417. 1909. TYPE: Mexico, Sonora, near Horcasitas, *Poselger* (not preserved). NEOTYPE (designed by Zappi, Succ. Pl. Res. 3: 147. 1994): *E.Palmer 362* (neotype: US 00115543!; isoneotypes: NY 00120557!, CM 1478!, K 000062714!). Note: this neotype designated by Zappi is the type of *Cephalocereus palmeri* (see below).
   = *Cereus cometes* Scheidw., Allg. Gartenzeitung (Otto & Dietrich) 8: 339. 1840. ≡ *Pilocereus cometes* Mittl. ex C.F.Först., Handb. Cacteenk. [Förster] 357. 1846. ≡ *Pilocereus jubatus* Salm-Dyck, Cact. Hort. Dyck. ed. I. 24; Lem. in Rev. Hortic. 427. 1862. ≡ *Cephalocereus cometes* Britton & Rose, Contr. U.S. Natl. Herb. 12: 416. 1909. ≡ *Pilosocereus cometes* (Scheidw.) Byles & G.D.Rowley, Cact. Succ. J. Gr. Brit. 19: 66. 1957. TYPE: Mexico, [San Luis] Potosí, [*Galeotti*?] (not preserved).
   = *Cephalocereus maxonii* Rose, Contr. U.S. Natl. Herb. 12: 417. 1909. ≡ *Cereus maxonii* Vaupel, Monatsschr. Kakteenk. 23: 23, 26. 1913. ≡ *Pilocereus maxonii* A.Berger, Kakteen (Berger) 345. 1929. ≡ *Pilosocereus maxonii* (Rose) Byles & G.D.Rowley, Cact. Succ. J. Gr. Brit. 19: 67. 1957. TYPE: Guatemala, Jalapa, near El Rancho, 4 Apr 1905, *W.R.Maxon* & *R.H.Hay 3769* (holotype: US 00115542!).
   = *Cephalocereus palmeri* Rose, Contr. U.S. Natl. Herb. 12: 418. 1909. ≡ *Cereus victoriensis* Vaupel, Monatsschr. Kakteenk. 23: 24, 37. 1913. ≡ *Pilocereus palmeri* (Rose) F.M.Knuth, Kaktus-ABC [Backeb. & Knuth] 333. 1936. ≡ *Pilosocereus palmeri* (Rose) Byles & G.D.Rowley, Cact. Succ. J. Gr. Brit. 19: 67. 1957. ≡ *Pilosocereus palmeri* var. *victoriensis* (Vaupel) Backeb., Kakteenlexikon 367. 1966. ≡ *Cephalophorus palmeri* (Rose) Boom, Succulenta (Netherlands) 46: 107. 1967. ≡ *Pilosocereus leucocephalus* subsp. *palmeri* (Rose) Scheinvar, Fl. Cactológ. Est. Querétaro 192. 2004. TYPE: Mexico, Tamaulipas, near Victoria, 320 m, 01 May–13 June 1907, *E.Palmer 362* (holotype: US 00115543!; isotypes: NY 00120557!, CM 1478!, K 000062714!).
   = *Cephalocereus sartorianus* Rose, Contr. U.S. Natl. Herb. 12: 419. 1909. ≡ *Cereus sartorianus* (Britton & Rose) Kupper ex A.Berger, Kakteen (Berger) 157. 1929. ≡ *Pilocereus sartorianus* (Britton & Rose) A.Berger, Kakteen (Berger) 345. 1929. ≡ *Pilosocereus sartorianus* (Rose) Byles & G.D.Rowley, Cact. Succ. J. Gr. Brit. 19: 69. 1957. ≡ *Pilosocereus palmeri* var. *sartorianus* (Rose) Lodé, Fichier Encycl. Cact. Autres Succ. 19: 1776. 1997. TYPE: Mexico, Veracruz, 1908, *C.A.Purpus s.n.* (holotype: US 00115545!).
6. ***Pilosocereus purpusii*** (Britton & Rose) Byles & G.D.Rowley, Cact. Succ. J. Gr. Brit. 19: 67. 1957. ≡ *Cephalocereus purpusii* Britton & Rose, Cactaceae (Britton & Rose) 2: 56. 1920. ≡ *Pilocereus purpusii* (Britton & Rose) F.M.Knuth, Kaktus-ABC [Backeb. & Knuth] 333. 1936. LECTOTYPE (designed by Zappi, Succ. Pl. Res. 3: 150. 1994): Mexico, Sinaloa, Mazatlán, near the town overlooking the sea, 31 Mar 1910, *J.N.Rose*, *P.C.Standley* & *P.G.Russell 13749* (lectotype: US 00115544!; isolectotype: NY 00120558!).
   = *Pilocereus guerreronis* Backeb., Beitr. Sukkulentenk. Sukkulentenpflege 1: 3. 1941. ≡ *Pilosocereus guerreronis* (Backeb.) Byles & G.D.Rowley, Cact. Succ. J. Gr. Brit. 19: 67. 1957. ≡ *Cephalocereus guerreronis* (Backeb.) Buxb., Bot. Stud. 12: 101. 1961. TYPE: Mexico, Guerrero, Cañón del Zopilote, 800 m (not preserved). LECTOTYPE (designed by Zappi, Succ. Pl. Res. 3: 144. 1994): Backeberg, in ibid.: 4, photo. 1941. **Synon. nov.**

In previous works, *P. guerreronis* had been assumed to be a local form of the widespread *P. alensis* (Anderson 2001; Hunt et al. 2006), and in some cases had been included in *P. alensis* with a question mark (Korotkova et al. 2021; Zappi 1994). Moreover, its morphological traits of branches and fertile region show differences with respect to *P. alensis*. Herein the name *P. guerreronis* is recognized as a synonym for *P. purpusii* as by examining the protologue of *P. guerreronis* (Backeberg 1941) its description and distribution is more consistent with our recognition of *P. purpusii* by presenting branches of 7 cm diameter, ribs of 14 mm height, distance between areoles of 15 mm, discontinuous fertile region, and whitish flowers.

### Species key to *Pilosocereus leucocephalus* group *s.s.*


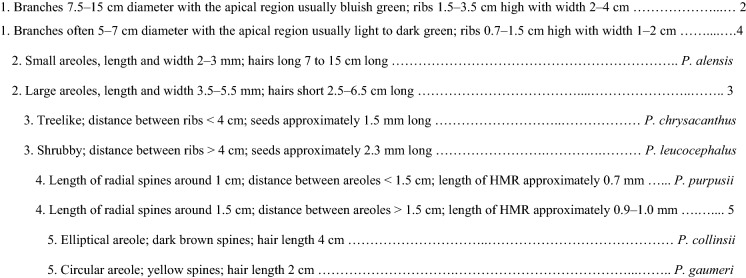


## Supplementary Information

Below is the link to the electronic supplementary material.

Supplementary file1 (PDF 1031 KB)

## References

[CR1] Akoglu H (2018). User’s guide to correlation coefficients. Turk J Emerg Med.

[CR2] Alvarado-Sizzo H, Casas A, Parra F, Arreola-Nava HJ, Terrazas T, Sánchez C (2018). Species delimitation in the *Stenocereus griseus* (Cactaceae) species complex reveals a new species *S. huastecorum*. PLoS ONE.

[CR3] Anderson EF (2001). The cactus family.

[CR4] Arroyo-Cosultchi G, Terrazas T, Arias S, Arreola-Nava HJ (2006). The systematic significance of seed morphology in *Stenocereus* (Cactaceae). Taxon.

[CR5] Backeberg C (1941). Die Kakteen des Zopilote-Cañons (Geierschlucht) in Guerrero (Mexico). Beitr Sukkulentenk Sukkulentenpflege.

[CR6] Bagheri A, Maassoumi AA, Rahiminejad MR (2017). Molecular phylogeny and divergence times of *Astragalus* section *Hymenostegis*: an analysis of a rapidly diversifying species group in Fabaceae. Sci Rep.

[CR7] Baker MA (2016). Morphological and cytological analyses in *Cylindropuntia* (Cactaceae): the taxonomic circumscription of *C. echinocarpa*, *C. multigeniculata*, and *C. whipplei*. J Bot Res Inst Tex.

[CR8] Baker MA, Johnson RA (2000). Morphometric analysis of *Escobaria sneedii* var. *sneedii*, *E. sneedii* var. *leei*, and *E. guadalupensis* (Cactaceae). Syst Bot.

[CR9] Barthlott W, Burstedde K, Geffert J (2015). Biogeography and biodiversity of cacti. Schumannia.

[CR10] Bravo-Hollis H (1978). Las cactáceas de México.

[CR11] Britton NL, Rose JN (1920). The Cactaceae: descriptions and illustrations of plants of the cactus family.

[CR12] Bustamante E, Búrquez A, Scheinvar E, Eguiarte LE (2016). Population genetic structure of a widespread bat-pollinated columnar cactus. PLoS ONE.

[CR13] Byles RS, Rowley GD (1957). *Pilosocereus* Byl. & Rowl. nom. gen. nov. (Cactaceae). Cact Succ J Gr Brit.

[CR14] Calonje M, Meerow AW, Griffith MP (2019). A time-calibrated species tree phylogeny of the New World cycad genus *Zamia* L. (Zamiaceae, Cycadales). Int J Plant Sci.

[CR15] Calvente A, Moraes EM, Lavor P (2016). Phylogenetic analyses of *Pilosocereus* (Cactaceae) inferred from plastid and nuclear sequences. Bot J Linn Soc.

[CR16] Chavent M, Kuentz-Simonet V, Labenne A, Saracco J (2017) Multivariate analysis of mixed data: the R package PCAmixdata. http://arxiv.org/abs/14114911

[CR17] Chen J, Wu G, Shrestha N (2021). Phylogeny and species delimitation of Chinese *Medicago* (Leguminosae) and its relatives based on molecular and morphological evidence. Front Plant Sci.

[CR18] Darriba D, Taboada GL, Doallo R, Posada D (2012). jModelTest 2: more models, new heuristics and parallel computing. Nat Methods.

[CR41] de Mendiburu F (2013) Statistical procedures for agricultural research. Package “agricolae”. CRAN-R. The R Foundation, Vienna

[CR19] de Queiroz K, Howard D, Berlocher S (1998). The general lineage concept of species, species criteria, and the process of speciation: a conceptual unification and terminological recommendations. Endless forms: species and speciation.

[CR20] Doyle JJ, Doyle JL (1987). A rapid DNA isolation procedure for small quantities of fresh leaf tissue. Phytochem Bull.

[CR21] Doyle ED, Prates I, Sampaio I (2021). Molecular phylogenetic inference of the howler monkey radiation (Primates: *Alouatta*). Primates.

[CR22] Duminil J, Di Michele M (2009). Plant species delimitation: a comparison of morphological and molecular markers. Plant Biosyst.

[CR23] Edgar RC (2004). Muscle: multiple sequence alignment with high accuracy and high throughput. Nucleic Acids Res.

[CR24] Farris JS, Källersjö M, Kluge AG, Bult C (1994). Testing significance of incongruence. Cladistics.

[CR25] Franck AR, Barrios D, Campbell KCSE (2019). Revision of *Pilosocereus* (Cactaceae) in the Caribbean and northern Andean region. Phytotaxa.

[CR26] Granados MC, Granados-Aguilar X, Donadío S (2017). Geographic structure in two highly diverse lineages of *Tillandsia* (Bromeliaceae). Botany.

[CR27] Granados-Aguilar X, Granados MC, Cervantes CR (2021). Unraveling reticulate evolution in *Opuntia* (Cactaceae) from southern Mexico. Front Plant Sci.

[CR28] Guzmán U, Arias S, Dávila P (2003). Catálogo de cactáceas mexicanas.

[CR29] Hernández-Canchola G, León-Paniagua L (2017). Genetic and ecological processes promoting early diversification in the lowland Mesoamerican bat *Sturnira parvidens* (Chiroptera: Phyllostomidae). Mol Phylogenet Evol.

[CR30] Hernández-Hernández T, Hernández HM, De-Nova JA (2011). Phylogenetic relationships and evolution of growth form in Cactaceae (Caryophyllales, Eudicotyledoneae). Am J Bot.

[CR31] Hunt D, Taylor N, Charles G (2006). The new cactus lexicon.

[CR32] Jiménez-Barron O, García-Sandoval R, Magallón S (2020). Phylogeny, diversification rate, and divergence time of *Agave sensu lato* (Asparagaceae), a group of recent origin in the process of diversification. Front Plant Sci.

[CR33] Korkmaz S, Goksuluk D, Zararsiz G (2014). MVN: an R package for assessing multivariate normality. R J.

[CR34] Korotkova N, Aquino D, Arias S (2021). Cactaceae at Caryophyllales.org—a dynamic online species-level taxonomic backbone for the family. Willdenowia.

[CR35] Lavor P, Calvente A, Versieux LM, Sanmartin I (2018). Bayesian spatio-temporal reconstruction reveals rapid diversification and Pleistocene range expansion in the widespread columnar cactus *Pilosocereus*. J Biogeogr.

[CR36] Lavor P, Versieux LM, Calvente A (2020). Phylogenetic relationships of *Pilosocereus* (Cactaceae) and taxonomic implications. PlantNow.

[CR37] Leaché AD, Koo MS, Spencer CL, Papenfuss TJ, Fisher RN, McGuire JA (2009). Quantifying ecological, morphological, and genetic variation to delimit species in the coast horned lizard species complex (*Phrynosoma*). Proc Natl Acad Sci.

[CR38] Lemus-Barrios H, Barrios D, García-Beltrán JA (2021). Taxonomic implications of seed morphology in *Melocactus* (Cactaceae) from Cuba. Willdenowia.

[CR39] Lendel A (2013) South American cacti in time and space: studies on the diversification of the tribe Cereeae, with particular focus on subtribe Trichocereinae (Cactaceae). Dissertation, Mathematisch-naturwissenschaftlichen Fakultät der Universität Zürich

[CR40] Lewis PO (2001). A likelihood approach to estimating phylogeny from discrete morphological character data. Syst Biol.

[CR42] Meudt HM, Prebble JM, Lehnebach CA (2015). Native New Zealand forget-me-nots (*Myosotis*, Boraginaceae) comprise a Pleistocene species radiation with very low genetic divergence. Plant Syst Evol.

[CR43] Moraes E, Perez M, Téo M, Zappi D, Taylor N, Machado M (2012). Cross-species amplification of microsatellites reveals incongruence in the molecular variation and taxonomic limits of the *Pilosocereus aurisetus* group (Cactaceae). Genetica.

[CR44] Morrone JJ (2014). Biogeographical regionalisation of the Neotropical region. Zootaxa.

[CR45] Müller J, Müller K, Quandt D, Neinhuis C (2005) PhyDE, phylogenetic data editor v.0.995. http://www.phyde.de/. Accessed 28 Jul 2020

[CR46] Pessoa EM, Alves M, Alves-Araújo A, Palma-Silva C, Pinheiro F (2012). Integrating different tools to disentangle species complexes: a case study in *Epidendrum* (Orchidaceae). Taxon.

[CR47] Piedra-malagón EM, Albarrán-lara AL, Rull J, Piñero D, Sosa V (2016). Using multiple sources of characters to delimit species in the genus *Crataegus* (Rosaceae): the case of the *Crataegus rosei* complex. Syst Biodivers.

[CR48] Pinheiro F, Dantas-Queiroz MV, Palma-Silva C (2018). Plant species complexes as models to understand speciation and evolution: a review of South American studies. Crit Rev Plant Sci.

[CR49] R Core Team (2013) R: a language and environment for statistical computing. http://www.r-project.org/. Accessed 29 Nov 2019

[CR50] Rambaut A (2014) FigTree. http://tree.bio.ed.ac.uk/software/figtree/. Accessed 28 Jul 2020

[CR51] Ripley B, Venables B, Bates DM et al (2013) Package “MASS”. CRAN-R. The R Foundation, Vienna

[CR52] Rivera-Lugo M, García-Mendoza A, Simpson J, Solano E, Gil-Vega K (2018). Taxonomic implications of the morphological and genetic variation of cultivated and domesticated populations of the *Agave angustifolia* complex (Agavoideae, Asparagaceae) in Oaxaca, Mexico. Plant Syst Evol.

[CR53] Ronquist F, Teslenko M, van der Mark P (2012). MrBayes 3.2: efficient bayesian phylogenetic inference and model choice across a large model space. Syst Biol.

[CR54] Ronquist F, Huelsenbeck J, Teslenko M (2011) MrBayes version 3.2 manual: tutorials and model summaries. http://nbisweden.github.io/MrBayes/manual.html. Accessed 28 Jul 2020

[CR55] Sánchez D, Gómez-Quintero D, Vargas-Ponce O (2020). Species delimitation in the *Echinocereus pulchellus* complex (Cactaceae). Brittonia.

[CR56] Schlumpberger BO, Renner SS (2012). Molecular phylogenetics of *Echinopsis* (Cactaceae): polyphyly at all levels and convergent evolution of pollination modes and growth forms. Am J Bot.

[CR57] Shaw J, Lickey EB, Schilling EE, Small RL (2007). Comparison of whole chloroplast genome sequences to choose noncoding regions for phylogenetic studies in angiosperms: the tortoise and the hare III. Am J Bot.

[CR58] Simmons MP, Ochoterena H (2000). Gaps as characters in sequence-based phylogenetic analyses. Syst Biol.

[CR59] Stamatakis A (2014). RAxML version 8: a tool for phylogenetic analysis and post-analysis of large phylogenies. Bioinformatics.

[CR60] Su X, Wu G, Li L, Liu J (2015). Species delimitation in plants using the Qinghai-Tibet Plateau endemic *Orinus* (Poaceae: Tridentinae) as an example. Ann Bot.

[CR61] Swofford DL (2003). PAUP*: phylogenetic analysis using parsimony (* and other methods). Version 4.

[CR62] Taberlet P, Gielly L, Pautou G, Bouvet J (1991). Universal primers for amplification of three non-coding regions of chloroplast DNA. Plant Mol Biol.

[CR63] Valencia-A S (2020). Species delimitation in the genus *Quercus* (Fagaceae). Bot Sci.

[CR64] Vázquez-Sánchez M, Terrazas T, Arias S (2012). El hábito y la forma de crecimiento en la tribu Cacteae (Cactaceae, Cactoideae). Bot Sci.

[CR65] Wei T, Simko V (2017) Package “corrplot”: visualization of a correlation matrix. CRAN-R. The R Foundation, Vienna

[CR66] Yetman D (2007). The great cacti: ethnobotany and biogeography.

[CR67] Zappi DC (1994). *Pilosocereus* (Cactaceae): the genus in Brazil. Suc Plant Res.

