# Supplementary material for: Phylogenetic and morphological analyses of Pilosocereus leucocephalus group s.s. (Cactaceae) reveal new taxonomical implications
Source: J Plant Res. 2022 Mar 19;135(3):423–42. doi: 10.1007/s10265-022-01384-x (PMC9081079; doi:10.1007/s10265-022-01384-x)
Supplement: Supplementary file 1 — Supplementary file1 (PDF 1031 KB) [file 10265_2022_1384_MOESM1_ESM.pdf]

SUPPLEMENTARY INFORMATION

Title:

Phylogenetic and morphological analyses of *Pilosocereus leucocephalus* group *s.s.* (Cactaceae) reveal new taxonomical implications

Journal:

Journal of Plant Research

Authors:

Daniel Franco-Estrada[1,2]*, Duniel Barrios[3], Cristian R. Cervantes[1,2], Xochitl Granados-Aguilar[1,2] and Salvador Arias[2]*

[1]Posgrado en Ciencias Biológicas, Instituto de Biología, Universidad Nacional Autónoma de México, Mexico City, Mexico.

[2]Jardín Botánico, Instituto de Biología, Universidad Nacional Autónoma de México, Mexico City, Mexico.

[3]Grupo de Ecología y Conservación, Jardín Botánico Nacional, Universidad de la Habana, Havana, Cuba.

*Authors for correspondence (daniel.franco@st.ib.unam.mx, sarias@ib.unam.mx)

Content:

Tables S1–S5

Fig. S1

Appendices S1 and S2

Table S1. Mean (mm) and standard deviation values for branch morphological characters of *Pilosocereus leucocephalus* group *s.s.* taxa. Italic characters indicate characters used in multivariate analysis with Pearson's correlation coefficient less than 0.6

Species	ID	Collector and number	Locality	*Branch diameter*	*Length of the longest radial spine*	Rib height	Rib width	Rib distance	*Rib width-distance ratio*	*Areole length*	Areole width	*Areole length-width ratio*	*Distance between areoles*	*Length of the longest hairs*
P. alensis	1	*DFE 55*	Trinidad García de la Cadena, Zac.	110.71 ± 6.75	13.55 ± 1.22	20.23 ± 0.88	25.59 ± 3.73	37.45 ± 1.53	0.68 ± 0.08	2.37 ± 0.24	2.36 ± 0.19	1.01 ± 0.11	11.92 ± 0.78	102.31 ± 9.47
P. alensis	2	*DFE 61*	Yonora, Mezquital, Dgo.	105.67 ± 3.25	13.32 ± 1.77	19.20 ± 1.69	23.34 ± 1.91	34.81 ± 2.32	0.67 ± 0.04	2.42 ± 0.16	2.40 ± 0.15	1.01 ± 0.09	11.08 ± 0.69	121.86 ± 12.43
P. alensis	3	*DFE 62*	El Zapote, Pueblo Nuevo, Dgo.	110.38 ± 4.21	12.43 ± 0.92	19.98 ± 1.60	22.86 ± 2.49	35.50 ± 4.37	0.65 ± 0.11	2.42 ± 0.19	2.33 ± 0.16	1.04 ± 0.12	12.19 ± 1.12	73.70 ± 8.26
P. alensis	4	*MG 4464*	Huahuapan, San Dimas, Dgo.	81.24 ± 1.18	12.94 ± 1.14	16.08 ± 0.88	21.01 ± 0.45	25.57 ± 1.03	0.82 ± 0.03	2.28 ± 0.27	2.28 ± 0.23	1.01 ± 0.06	12.06 ± 1.08	87.72 ± 9.23
P. chrysacanthus	5	*DFE 32*	Santa Cruz, Ajalpan, Pue.	99.47 ± 9.32	13.24 ± 2.43	19.82 ± 1.12	22.08 ± 2.54	30.93 ± 1.76	0.71 ± 0.06	4.30 ± 0.23	4.34 ± 0.22	0.99 ± 0.06	16.39 ± 2.89	31.17 ± 4.93
P. chrysacanthus	6	*DFE 34*	Loma Pelada, Santa María Tecomavaca, Oax.	98.70 ± 2.67	15.71 ± 1.66	24.15 ± 1.53	24.37 ± 1.51	40.51 ± 1.16	0.61 ± 0.04	4.30 ± 0.40	4.25 ± 0.32	1.01 ± 0.07	16.60 ± 2.76	26.75 ± 3.19
P. chrysacanthus	7	*DFE 35*	San Juan Bautista Cuicatlán, Oax.	109.27 ± 6.43	13.40 ± 1.53	23.05 ± 1.46	25.24 ± 4.38	38.63 ± 1.55	0.65 ± 0.12	4.36 ± 0.24	4.14 ± 0.27	1.05 ± 0.09	16.26 ± 1.11	31.68 ± 4.59
P. chrysacanthus	8	*DFE 36*	Santiago Dominguillo, San Juan Bautista Atatlahuca, Oax.	92.06 ± 2.64	14.85 ± 2.03	26.69 ± 1.45	31.60 ± 1.74	38.08 ± 1.46	0.83 ± 0.06	4.30 ± 0.34	4.12 ± 0.39	1.05 ± 0.10	17.56 ± 1.01	34.28 ± 4.33
P. chrysacanthus	9	*DFE 42*	Nuevos Horizontes, Acatlán, Pue.	105.51 ± 9.42	14.82 ± 1.35	22.37 ± 1.92	26.33 ± 0.73	35.25 ± 1.99	0.74 ± 0.04	3.55 ± 0.22	3.31 ± 0.16	1.07 ± 0.06	18.19 ± 2.02	35.39 ± 3.61
P. chrysacanthus	10	*YM 03*	Santiago Huauclilla, Oax.	96.40 ± 11.42	15.93 ± 2.81	18.66 ± 0.85	29.25 ± 2.82	33.93 ± 2.13	0.86 ± 0.07	4.30 ± 0.37	4.14 ± 0.29	1.04 ± 0.10	16.91 ± 2.52	30.16 ± 2.71
P.collinsii	11	*DFE 39*	Cerro Guiengola, Santo Domingo Tehuantepec, Oax.	55.33 ± 6.96	14.20 ± 1.87	8.59 ± 0.96	15.06 ± 1.53	17.58 ± 1.25	0.86 ± 0.13	4.47 ± 0.21	3.18 ± 0.23	1.41 ± 0.11	21.12 ± 1.29	20.67 ± 1.86
P.collinsii	12	*DFE 65*	Los Ciruelos, San Pedro Pochutla, Oax.	49.50 ± 3.02	13.18 ± 1.64	11.03 ± 1.05	11.52 ± 0.51	17.21 ± 1.11	0.67 ± 0.04	3.73 ± 0.40	2.76 ± 0.29	1.36 ± 0.21	22.72 ± 2.45	39.06 ± 3.39
P.collinsii	13	*DFE 66*	El Coyul, San Pedro Huamelula, Oax.	53.87 ± 5.08	12.17 ± 1.17	10.70 ± 1.06	12.39 ± 1.23	16.66 ± 1.65	0.74 ± 0.05	4.06 ± 0.35	2.47 ± 0.22	1.64 ± 0.12	17.82 ± 1.07	42.54 ± 6.26
P.collinsii	14	*DFE 67*	Playa Grande, San Pedro Huamelula, Oax.	51.63 ± 3.51	10.97 ± 0.95	10.08 ± 1.43	13.23 ± 2.14	20.43 ± 1.43	0.64 ± 0.07	3.82 ± 0.60	2.56 ± 0.37	1.49 ± 0.17	20.79 ± 2.52	43.51 ± 5.64
P.collinsii	15	*DFE 68*	Santa María Tonameca, Oax.	56.97 ± 5.92	12.43 ± 1.28	13.67 ± 0.78	14.60 ± 2.45	21.24 ± 2.37	0.68 ± 0.07	4.75 ± 0.35	3.36 ± 0.25	1.42 ± 0.15	18.74 ± 1.33	46.90 ± 5.83
P.collinsii	16	*DFE 69*	Bahía El Maguey, Santa María Huatulco, Oax.	48.79 ± 1.26	12.67 ± 1.29	10.17 ± 0.80	11.76 ± 0.69	16.09 ± 1.61	0.74 ± 0.11	3.96 ± 0.43	2.75 ± 0.48	1.46 ± 0.16	19.23 ± 1.38	54.54 ± 5.76

Table S1. Mean (mm) and standard deviation values for branch morphological characters of *Pilosocereus leucocephalus* group *s.s.* taxa. Italic characters indicate characters used in multivariate analysis with Pearson's correlation coefficient less than 0.6

Species	ID	Collector and number	Locality	*Branch diameter*	*Length of the longest radial spine*	Rib height	Rib width	Rib distance	*Rib width-distance ratio*	*Areole length*	Areole width	*Areole length-width ratio*	Distance between areoles	*Length of the longest hairs*
P. cometes	17	*DFE 47*	Arroyo Seco, Qro.	108.08 ± 8.40	14.69 ± 2.24	25.79 ± 1.27	30.12 ± 2.13	42.80 ± 3.37	0.71 ± 0.09	4.36 ± 0.25	4.40 ± 0.27	0.99 ± 0.08	18.89 ± 1.61	43.05 ± 8.11
P. cometes	18	*DFE 48*	Vaqueros, Lagunillas, S.L.P.	119.81 ± 3.78	17.01 ± 2.15	28.55 ± 1.07	29.64 ± 2.27	46.01 ± 2.67	0.64 ± 0.03	4.32 ± 0.24	4.34 ± 0.25	0.99 ± 0.07	16.84 ± 1.83	52.28 ± 12.18
P. gaumeri	19	*DFE 70*	Ucú, Yuc.	48.51 ± 3.21	14.19 ± 1.14	11.26 ± 1.69	12.39 ± 0.68	17.62 ± 1.04	0.70 ± 0.06	3.42 ± 0.34	3.04 ± 0.33	1.12 ± 0.08	18.86 ± 1.46	21.55 ± 3.24
P. gaumeri	20	*DFE 71*	Dzemul, Yuc.	51.44 ± 2.64	14.38 ± 1.04	11.19 ± 0.71	13.69 ± 2.01	19.52 ± 1.41	0.69 ± 0.07	3.41 ± 0.27	3.28 ± 0.23	1.04 ± 0.09	18.43 ± 0.99	18.97 ± 1.67
P. gaumeri	21	*DFE 72*	Progreso, Yuc.	48.03 ± 4.17	12.71 ± 0.78	11.14 ± 1.04	14.06 ± 1.01	16.33 ± 1.53	0.86 ± 0.06	3.10 ± 0.28	2.89 ± 0.28	1.07 ± 0.11	15.59 ± 1.75	23.50 ± 4.52
P. gaumeri	22	*DFE 73*	Tecoh, Yuc.	52.77 ± 5.39	13.20 ± 1.41	10.50 ± 0.60	11.08 ± 0.70	16.21 ± 0.39	0.68 ± 0.04	2.57 ± 0.31	2.22 ± 0.33	1.16 ± 0.10	17.90 ± 2.59	17.41 ± 2.28
P. gaumeri	23	*DFE 74*	Cuzamá, Yuc.	41.71 ± 5.59	12.86 ± 1.11	10.01 ± 0.96	10.63 ± 0.83	17.11 ± 1.01	0.62 ± 0.06	2.61 ± 0.29	2.48 ± 0.30	1.05 ± 0.11	17.76 ± 1.22	18.87 ± 2.79
P. leucocephalus	24	*DFE 41*	Abelardo L. Rodríguez, Cintalapa, Chis.	119.01 ± 5.70	16.89 ± 2.98	24.39 ± 2.17	31.45 ± 1.70	43.95 ± 1.67	0.71 ± 0.03	5.21 ± 0.30	5.25 ± 0.28	0.99 ± 0.07	19.56 ± 1.77	39.16 ± 9.59
P. leucocephalus	25	*DFE 43*	Xalapa, Ver.	95.31 ± 5.77	13.57 ± 2.46	28.33 ± 2.31	32.83 ± 1.04	41.34 ± 2.68	0.79 ± 0.05	4.34 ± 0.46	4.34 ± 0.59	1 ± 0.08	17.71 ± 2.08	57.39 ± 11.45
P. leucocephalus	26	*DFE 44*	Cerro Gordo, Emiliano Zapata, Ver.	110.69 ± 12.31	13.79 ± 1.47	30.94 ± 1.95	33.61 ± 2.60	47.95 ± 3.54	0.70 ± 0.07	4.57 ± 0.39	4.55 ± 0.36	1 ± 0.09	17.44 ± 1.64	58.75 ± 11.51
P. leucocephalus	27	*DFE 45*	Pinoltepec, Emiliano Zapata, Ver.	103.93 ± 15.19	14.45 ± 2.28	25.35 ± 2.43	34.41 ± 1.31	46.26 ± 2.83	0.74 ± 0.04	4.24 ± 0.22	4.24 ± 0.31	1 ± 0.07	17.94 ± 2.83	53.89 ± 6.48
P. leucocephalus	28	*DFE 46*	San Isidro, Puente Nacional, Ver.	122.71 ± 6.44	17.45 ± 2.20	31.74 ± 2.53	36.59 ± 6.48	49.40 ± 2.52	0.73 ± 0.11	4.41 ± 0.29	4.52 ± 0.23	0.97 ± 0.08	21.03 ± 1.82	50.94 ± 3.94
P. leucocephalus	29	*DFE 58*	Cañon de la Libertad, Victoria, Tamps.	139.14 ± 5.82	16.11 ± 1.83	29.80 ± 1.59	37.81 ± 2.57	48.15 ± 3.51	0.78 ± 0.05	4.49 ± 0.23	4.50 ± 0.22	1 ± 0.06	21.30 ± 1.61	62.05 ± 9.05
P. leucocephalus	30	*DFE 59*	San Antonio, Victoria, Tamps.	105.43 ± 8.13	18.85 ± 1.92	29.35 ± 2.54	35.69 ± 3.62	40.81 ± 2.96	0.87 ± 0.07	4.52 ± 0.27	4.50 ± 0.29	1 ± 0.08	21.12 ± 2.84	60.50 ± 6.12
P. leucocephalus	31	*DFE 60*	Los Nogales, Jaumave, Tamps.	95.52 ± 5.84	17.06 ± 2.47	24.95 ± 1.57	24.79 ± 1.30	39.07 ± 4.01	0.63 ± 0.05	4.47 ± 0.30	4.34 ± 0.25	1.03 ± 0.07	18.06 ± 2.81	53.94 ± 5.49
P. purpusii	32	*DFE 50*	Laguna Cuyutlán, Manzanillo, Col.	85.51 ± 3.75	9.09 ± 1.08	13.86 ± 0.71	18.77 ± 0.83	24.16 ± 1.46	0.77 ± 0.04	2.91 ± 0.14	2.86 ± 0.16	1.02 ± 0.07	10.49 ± 1.11	36.38 ± 4.97
P. purpusii	33	*DFE 51*	La Huerta, Jal.	82.49 ± 3.86	11.58 ± 1.02	14.51 ± 1.19	17.76 ± 1.41	24.69 ± 2.99	0.72 ± 0.05	2.87 ± 0.18	2.95 ± 0.17	0.97 ± 0.08	12.99 ± 1.23	36.29 ± 4.12
P. purpusii	34	*DFE 53*	Chamela, La Huerta, Jal.	67.66 ± 2.49	10.56 ± 0.77	12.75 ± 1.06	16.15 ± 1.16	20.21 ± 1.53	0.80 ± 0.03	2.73 ± 0.24	2.76 ± 0.27	0.99 ± 0.07	11.28 ± 0.52	53.70 ± 5.70
P. purpusii	35	*DFE 54*	Manantlán, Autlán de Navarro, Jal.	80.58 ± 3.37	11.06 ± 1.21	10.96 ± 0.75	16.27 ± 0.37	19.81 ± 0.81	0.82 ± 0.04	2.70 ± 0.23	2.54 ± 0.22	1.06 ± 0.06	11.44 ± 0.54	54.03 ± 5.93
P. purpusii	36	*DFE 63*	Estero del Yugo, Mazatlán, Sin.	61.50 ± 2.01	9.92 ± 0.91	8.45 ± 0.73	15.42 ± 0.95	19.26 ± 0.78	0.80 ± 0.06	2.86 ± 0.18	2.45 ± 0.19	1.17 ± 0.08	13.81 ± 1.55	32.24 ± 3.70

Table S1. Mean (mm) and standard deviation values for branch morphological characters of *Pilosocereus leucocephalus* group *s.s.* taxa. Italic characters indicate characters used in multivariate analysis with Pearson's correlation coefficient less than 0.6

Species	ID	Collector and number	Locality	*Branch diameter*	*Length of the longest radial spine*	Rib height	Rib width	Rib distance	*Rib width-distance ratio*	*Areole length*	Areole width	*Areole length-width ratio*	*Distance between areoles*	*Length of the longest hairs*
P. quadricentralis	37	*DFE 37*	San Pedro Totolapam, Oax.	85.40 ± 4.61	11.27 ± 1.07	15.51 ± 0.85	19.70 ± 2.13	25.03 ± 1.45	0.78 ± 0.08	4.82 ± 0.56	4.44 ± 0.35	1.08 ± 0.12	14.42 ± 1.93	35.97 ± 5.12
P. quadricentralis	38	*DFE 38*	San José de Gracia, San Pedro Totolapa, Oax.	78.38 ± 3.07	13.42 ± 1.40	16.16 ± 0.76	20.37 ± 0.94	26.88 ± 1.43	0.75 ± 0.05	4.82 ± 0.30	4.34 ± 0.25	1.11 ± 0.10	15.16 ± 1.18	38.62 ± 4.56
P. quadricentralis	39	*DFE 64*	Barranca del Murciélago, Santa María Zoquitlán, Oax.	86.94 ± 7.18	12.73 ± 1.41	15.84 ± 1.36	18.79 ± 0.72	25.41 ± 1.78	0.74 ± 0.03	4.86 ± 0.44	3.99 ± 0.30	1.22 ± 0.11	14.22 ± 0.76	38.87 ± 4.76

Table S1. Mean (mm) and standard deviation values for flower and seed morphological characters of *Pilosocereus leucocephalus* group *s.s.* taxa. Italic characters indicate characters used in multivariate analysis with Pearson's correlation coefficient less than 0.6

Species	ID	Collector and number	Locality	Flower length	*Perianth width*	*Style length*	Sample size	Seed length	*Seed width*	*Seed length-width ratio*	*Hilum-micropylar region length*	*Hilum-micropylar region width*	Sample size
P. alensis	1	*DFE 55*	Trinidad García de la Cadena, Zac.	67.28 ± 8.08	28.09 ± 0.75	53.18 ± 11.92	03	–	–	–	–	–	–
P. alensis	2	*DFE 61*	Yonora, Mezquital, Dgo.	–	–	–	–	2.60 ± 0.14	1.77 ± 0.11	1.46 ± 0.04	1.11 ± 0.06	0.65 ± 0.09	20
P. alensis	3	*DFE 62*	El Zapote, Pueblo Nuevo, Dgo.	76.24 ± 3.89	33.90 ± 1.11	70.31 ± 6.61	04	2.34 ± 0.11	1.66 ± 0.07	1.41 ± 0.04	0.94 ± 0.07	0.47 ± 0.04	20
P. alensis	4	*MG 4464*	Huahuapan, San Dimas, Dgo.	–	–	–	–	–	–	–	–	–	–
P. chrysacanthus	5	*DFE 32*	Santa Cruz, Ajalpan, Pue.	93.94 ± 5.36	36.41 ± 4.72	75.17 ± 7.48	05	–	–	–	–	–	–
P. chrysacanthus	6	*DFE 34*	Loma Pelada, Santa María Tecomavaca, Oax.	–	–	–	–	1.92 ± 0.12	1.30 ± 0.06	1.46 ± 0.03	0.72 ± 0.09	0.36 ± 0.04	20
P. chrysacanthus	7	*DFE 35*	San Juan Bautista Cuicatlán, Oax.	–	–	–	–	2.09 ± 0.06	1.47 ± 0.07	1.42 ± 0.06	0.94 ± 0.04	0.46 ± 0.02	20
P. chrysacanthus	8	*DFE 36*	Santiago Dominguillo, San Juan Bautista Atatlahuca, Oax.	104.68 ± 4.75	46.27 ± 0.27	92.13 ± 0.15	04	1.98 ± 0.05	1.47 ± 0.05	1.34 ± 0.04	0.73 ± 0.05	0.43 ± 0.04	20
P. chrysacanthus	9	*DFE 42*	Nuevos Horizontes, Acatlán, Pue.	79.60 ± 3.23	44.26 ± 4.45	66.83 ± 7.74	05	1.96 ± 0.07	1.25 ± 0.05	1.56 ± 0.04	0.78 ± 0.08	0.45 ± 0.05	20
P. chrysacanthus	10	*YM 03*	Santiago Huauclilla, Oax.	–	–	–	–	–	–	–	–	–	–

Table S1. Mean (mm) and standard deviation values for flower and seed morphological characters of *Pilosocereus leucocephalus* group *s.s.* taxa. Italic characters indicate characters used in multivariate analysis with Pearson's correlation coefficient less than 0.6

Species	ID	Collector and number	Locality	Flower length	*Perianth width*	*Style length*	Sample size	Seed length	*Seed width*	*Seed length-width ratio*	*Hilum-micropylar region length*	*Hilum-micropylar region width*	Sample size
P.collinsii	11	*DFE 39*	Cerro Guiengola, Santo Domingo Tehuantepec, Oax.	71.85 ± 3.22	31.84 ± 2.09	62.65 ± 6.64	04	–	–	–	–	–	–
P.collinsii	12	*DFE 65*	Los Ciruelos, San Pedro Pochutla, Oax.	–	–	–	–	–	–	–	–	–	–
P.collinsii	13	*DFE 66*	El Coyul, San Pedro Huamelula, Oax.	68.94 ± 2.60	29.23 ± 4.22	61.81 ± 6.06	02	2.18 ± 0.06	1.52 ± 0.04	1.43 ± 0.02	1.05 ± 0.04	0.57 ± 0.03	20
P.collinsii	14	*DFE 67*	Playa Grande, San Pedro Huamelula, Oax.	61.85 ± 3.06	27.30 ± 1.40	53.27 ± 5.67	03	2.23 ± 0.05	1.50 ± 0.05	1.48 ± 0.03	0.94 ± 0.07	0.48 ± 0.03	20
P.collinsii	15	*DFE 68*	Santa María Tonameca, Oax.	–	–	–	–	2.09 ± 0.06	1.51 ± 0.05	1.38 ± 0.05	0.95 ± 0.04	0.46 ± 0.04	10
P.collinsii	16	*DFE 69*	Bahía El Maguey, Santa María Huatulco, Oax.	–	–	–	–	–	–	–	–	–	–
P. cometes	17	*DFE 47*	Arroyo Seco, Qro.	66.48 ± 7.82	32.33 ± 5.84	53.05 ± 7.22	09	2.13 ± 0.07	1.43 ± 0.05	1.49 ± 0.05	1.01 ± 0.07	0.46 ± 0.04	20
P. cometes	18	*DFE 48*	Vaqueros, Lagunillas, S.L.P.	64.41 ± 5.02	33.60 ± 2.74	57.16 ± 5.04	05	2.16 ± 0.08	1.36 ± 0.06	1.58 ± 0.01	0.93 ± 0.10	0.39 ± 0.01	02
P. gaumeri	19	*DFE 70*	Ucú, Yuc.	76.38 ± 4.50	24.41 ± 1.98	61.06 ± 5.82	03	–	–	–	–	–	–
P. gaumeri	20	*DFE 71*	Dzemul, Yuc.	50.77	28.88	40.68	01	1.92 ± 0.06	1.33 ± 0.04	1.44 ± 0.04	0.94 ± 0.07	0.43 ± 0.03	20
P. gaumeri	21	*DFE 72*	Progreso, Yuc.	47.96	28.91	39.23	01	1.87 ± 0.08	1.29 ± 0.06	1.45 ± 0.08	0.74 ± 0.04	0.37 ± 0.03	20
P. gaumeri	22	*DFE 73*	Tecoh, Yuc.	66.77 ± 12.07	26.57 ± 2.05	66.17 ± 11.07	03	2.36 ± 0.10	1.52 ± 0.06	1.55 ± 0.06	0.96 ± 0.05	0.51 ± 0.04	20
P. gaumeri	23	*DFE 74*	Cuzamá, Yuc.	68.92 ± 6.80	25.42 ± 3.47	63.72 ± 3.53	06	2.31 ± 0.11	1.61 ± 0.07	1.43 ± 0.07	0.84 ± 0.05	0.42 ± 0.05	20
P. leucocephalus	24	*DFE 41*	Abelardo L. Rodríguez, Cintalapa, Chis.	–	–	–	–	2.29 ± 0.06	1.59 ± 0.10	1.44 ± 0.08	0.92 ± 0.06	0.51 ± 0.04	20
P. leucocephalus	25	*DFE 43*	Xalapa, Ver.	–	–	–	–	2.11 ± 0.05	1.45 ± 0.08	1.45 ± 0.09	0.83 ± 0.04	0.41 ± 0.03	20
P. leucocephalus	26	*DFE 44*	Cerro Gordo, Emiliano Zapata, Ver.	–	–	–	–	1.92 ± 0.10	1.37 ± 0.06	1.40 ± 0.06	0.78 ± 0.06	0.42 ± 0.03	20
P. leucocephalus	27	*DFE 45*	Pinoltepec, Emiliano Zapata, Ver.	–	–	–	–	–	–	–	–	–	–
P. leucocephalus	28	*DFE 46*	San Isidro, Puente Nacional, Ver.	–	–	–	–	1.98 ± 0.07	1.39 ± 0.07	1.42 ± 0.05	0.82 ± 0.03	0.40 ± 0.02	20

Table S1. Mean (mm) and standard deviation values for flower and seed morphological characters of *Pilosocereus leucocephalus* group *s.s.* taxa. Italic characters indicate characters used in multivariate analysis with Pearson's correlation coefficient less than 0.6

Species	ID	Collector and number	Locality	Flower length	*Perianth width*	*Style length*	Sample size	Seed length	*Seed width*	*Seed length-width ratio*	*Hilum-micropylar region length*	*Hilum-micropylar region width*	Sample size
P. leucocephalus	29	*DFE 58*	Cañon de la Libertad, Victoria, Tamps.	72.90 ± 8.38	38.01 ± 4.46	56.85 ± 7.06	03	2.12 ± 0.09	1.49 ± 0.09	1.42 ± 0.08	0.87 ± 0.10	0.42 ± 0.05	20
P. leucocephalus	30	*DFE 59*	San Antonio, Victoria, Tamps.	73.82 ± 8.65	39.64 ± 4.18	61.39 ± 10.96	02	2.11 ± 0.10	1.39 ± 0.07	1.51 ± 0.09	0.87 ± 0.06	0.39 ± 0.03	20
P. leucocephalus	31	*DFE 60*	Los Nogales, Jaumave, Tamps.	66.12 ± 7.09	38.76 ± 4.87	52.72 ± 5.63	11	2.10 ± 0.06	1.46 ± 0.04	1.44 ± 0.04	0.86 ± 0.07	0.37 ± 0.02	20
P. purpusii	32	*DFE 50*	Laguna Cuyutlán, Manzanillo, Col.	66.34 ± 6.01	36.69 ± 2.88	58.28 ± 5.54	07	–	–	–	–	–	–
P. purpusii	33	*DFE 51*	La Huerta, Jal.	–	–	–	–	–	–	–	–	–	–
P. purpusii	34	*DFE 53*	Chamela, La Huerta, Jal.	56.89 ± 5.18	30.13 ± 2.47	52.17 ± 6.59	04	2.04 ± 0.08	1.35 ± 0.03	1.50 ± 0.02	0.83 ± 0.06	0.41 ± 0.01	03
P. purpusii	35	*DFE 54*	Manantlán, Autlán de Navarro, Jal.	59.26 ± 6.78	30.45 ± 2.93	55.14 ± 7.17	08	–	–	–	–	–	–
P. purpusii	36	*DFE 63*	Estero del Yugo, Mazatlán, Sin.	67.35 ± 8.45	28.67 ± 0.49	59.86 ± 5.26	02	1.99 ± 0.11	1.35 ± 0.07	1.48 ± 0.13	0.76 ± 0.04	0.37 ± 0.02	20
P. quadricentralis	37	*DFE 37*	San Pedro Totolapam, Oax.	–	–	–	–	1.76 ± 0.06	1.16 ± 0.06	1.52 ± 0.09	0.74 ± 0.04	0.33 ± 0.02	20
P. quadricentralis	38	*DFE 38*	San José de Gracia, San Pedro Totolapa, Oax.	76.26 ± 10.52	36.60 ± 4.49	62.47 ± 13.39	03	–	–	–	–	–	–
P. quadricentralis	39	*DFE 64*	Barranca del Murciélago, Santa María Zoquitlán, Oax.	66.81 ± 6.57	36.94 ± 3.15	51.35 ± 8.73	09	–	–	–	–	–	–

Table S2. Component loadings in the principal component analysis of quantitative characters in *Pilosocereus leucocephalus* group *s.s.* A = the case for 39 localities and B = the case for 14 localities. The characters with the highest loadings are highlighted in bold

Character	A		B	
	Component 1 (PC1)	Component 2 (PC2)	Component 1 (PC1)	Component 2 (PC2)
Branch diameter	0.31	**0.55**	**0.42**	0.11
Length of the longest radial spine	**0.58**	0.11	0.36	0.27
Rib width-distance ratio	–0.04	0.03	0.22	–0.21
Areole length	**0.54**	–0.15	0.32	0.38
Areole length-width ratio	–0.09	**–0.53**	–0.30	0.23
Distance between areoles	0.50	–0.38	0.12	**0.44**
Length of the longest hairs	–0.03	0.46	0.24	0.15
Perianth width	–	–	**0.42**	–0.04
Style length	–	–	0.03	–0.26
Seed width	–	–	–0.25	0.14
Seed length-width ratio	–	–	0.01	0.09
Hilum-micropylar region length	–	–	–0.19	**0.52**
Hilum-micropylar region width	–	–	–0.28	0.26
Percentage of the total variance explained	32.3	31.4	33.5	18.1

Table S3. Component loadings in the principal component analysis of quantitative and qualitative characters in *Pilosocereus leucocephalus* group *s.s.* A = the case for 39 localities and B = the case for 14 localities. The characters with the highest loadings are highlighted in bold

	A		B	
Character	Component 1 (PC1)	Component 2 (PC2)	Component 1 (PC1)	Component 2 (PC2)
Branch diameter	**0.87**	–0.07	**0.91**	–0.27
Length of the longest radial spine	0.54	0.32	0.74	–0.18
Rib width-distance ratio	–0.04	–0.01	0.39	0.08
Areole length	0.19	**0.69**	0.74	0.23
Areole length-width ratio	–0.57	0.54	–0.47	0.74
Distance between areoles	–0.07	**0.79**	0.31	0.33
Length of the longest hairs	0.74	–0.03	0.58	–0.15
Perianth width	–	–	**0.92**	0.12
Style length	–	–	0.14	0.39
Seed width	–	–	–0.43	0.05
Seed length-width ratio	–	–	–0.06	–0.26
Hilum-micropylar region length	–	–	–0.29	0.05
Hilum-micropylar region width	–	–	–0.39	0.61
Habit	0.27	0.57	0.73	**0.98**
Areole shape	0.17	0.46	0.28	**0.97**
Branch color at the apex	**0.75**	0.16	0.64	0.96
Spine color at the branch apex	0.35	**0.84**	**0.92**	**0.97**
Fertile part disposition	**0.81**	0.01	0.58	0.95
Percentage of the total variance explained	29	23.9	25.9	22.6

Table S4. Linear loadings in the linear discriminant analysis of seven quantitative characters in *Pilosocereus leucocephalus* group *s.s.* from 39 localities. The characters with the highest loadings are highlighted in bold

Character	Linear 1 (LD1)	Linear 2 (LD2)
Branch diameter	**–5.81**	–2.05
Length of the longest radial spine	–0.93	0.48
Rib width-distance ratio	–0.92	0.58
Areole length	2.33	**–8.95**
Areole length-width ratio	**9.30**	0.18
Distance between areoles	3.03	**–4.34**
Length of the longest hairs	–1.37	–1.04
Proportion of trace	0.46	0.33

Table S5. Information on the matrices used for phylogenetic inference in the present study

	rpl16	*trnL-trnF*	*petL-psbE*	*AT1G18270*	Concatenated matrix
Terminals	72	67	65	59	74
Length of aligned matrix	966	1,029	499	771	3,265
Constant characters	887	916	461	651	2,915
Variable characters	52	96	22	97	267
Informative characters	27	17	16	23	83
Percentage of informative characters	2.79	1.65	3.20	2.98	2.54
Nonalignable and excluded site (bp)	–	–	–	1 (156)	–
Molecular substitution model	F81 +G	F81 +G	F81	HKY +I	–
Indels	8	6	5	–	19

Fig. S1. Symmetric matrix of correlations calculated with Pearson's correlation coefficient for quantitative characters of *Pilosocereus leucocephalus* group *s.s.* Reference characters: A = branch diameter, B = length of the longest radial spines, C = rib height, D = rib width, E = rib distance, F = rib width-distance ratio, G = areole length, H = areole width, I = areole length-width ratio, J = distance between areoles, K = length of the longest hairs, L = flower length, M = perianth width, N = style length, O = seed length, P = seed width, Q = seed length-width ratio, R = hilum-micropylar region length, S = hilum-micropylar region width. Positive and negative correlations are indicated in the right bar with shades of blue and red, respectively

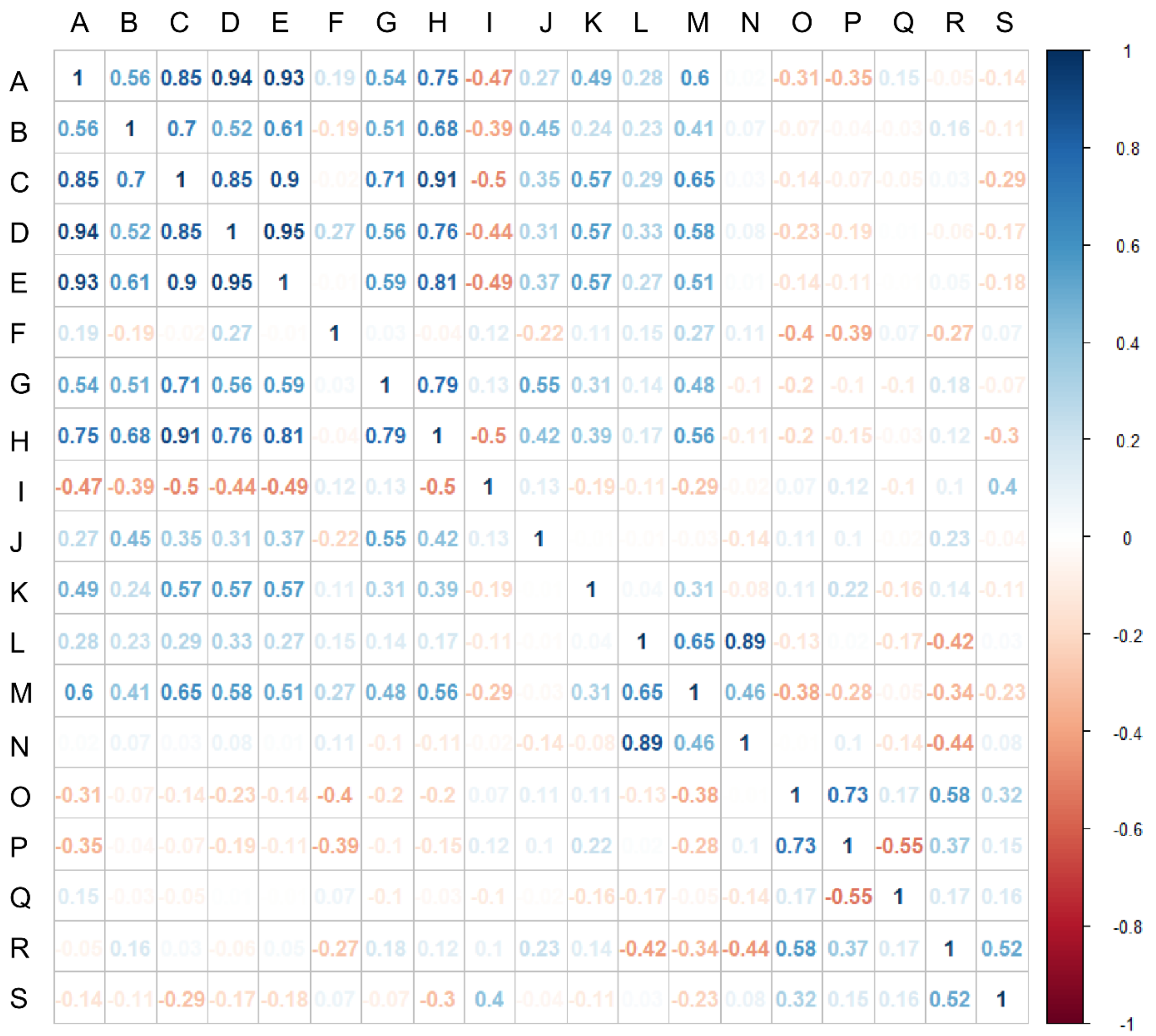

Appendix S1. List of taxa sampled, provenance, voucher (herbarium acronym) and accession number in GenBank. Country codes: AR Argentina, BR Brazil, CU Cuba, HN Honduras, MX Mexico, PE Peru, VE Venezuela, and SV El Salvador. The order of the sequences according to the accession number in GenBank is the following: *rpl16/ trnL-trnF/ petL-psbE/ AT1G18270*. The dash (–) indicates the missing sequence

1. ***Arrojadoa rhodantha*** Britton & Rose. BR, HM041385/ HM041230/ KX301129/ –. 2. ***Browningia hertlingiana*** (Backeb.) Buxb. PE, *B.O.Schlumpberger x101* (M) JQ779822/ JQ779688/ –/ –. 3. ***Cereus fernambucensis*** Lem. BR, KR998128/ KT164952/ KR998108/ –. 4. ***C. hildmannianus*** K. Schum. BR, KR998134/ HM041240/ KR998114/ –. 5. ***Lasiocereus fulvus*** F. Ritter. PE, Amazonas: *B.O.Schlumpberger x59* (M) JQ779821/ JQ779687/ –/ –. 6. ***Melocactus curvispinus*** Pfeiff. MX, Jalisco: *DFE 52* (MEXU) MZ541776/ MZ541719/ MZ541661/ MZ541602. 7. ***M. intortus*** (Mill.) Urb. DBG AZ, HM041455/ HM041301/ –/ –. 8. ***Pilosocereus alensis*** (F.A.C. Weber) Byles & G.D. Rowley. MX, Zacatecas: *DFE 55* (MEXU) MZ541777/ MZ541720/ MZ541662/ MZ541603. 9. ***P. alensis***. MX, Durango: *DFE 61* (MEXU) MZ541778/ MZ541721/ MZ541663/ MZ541604. 10. ***P. alensis***. MX, Durango: *DFE 62* (MEXU) MZ541779/ MZ541722/ MZ541664/ MZ541605. 11. ***P. alensis***. MX, Durango: *MG 4572* (CIIDIR) MZ541780/ MZ541723/ MZ541665/ MZ541606. 12. ***P. aureispinus*** (Buining & Brederoo) F. Ritter. BR, EM Moraes S21 (HUFS) –/ JN035538/ KX301123/ –. 13. ***P. brooksianus*** (Britton & Rose) Byles & G.D. Rowley. CU, Guantánamo: *Barrios et al*. HFC88773 (HAJB) MZ541781/ MZ541724/ MZ541666/ MZ541607. 14. ***P. brooksianus***. CU, Guantánamo: *Barrios et al*. HFC88773–2 (HAJB) MZ541782/ MZ541725/ MZ541667/ MZ541608. 15. ***P. brooksianus***. CU, Santiago de Cuba: *Barrios et al*. HFC88786 (HAJB) MZ541783/ MZ541726/ –/ –. 16. ***P. chrysacanthus*** (F.A.C. Weber ex Schum.) Byles & G.D. Rowley. MX, Puebla: *DFE 32* (MEXU) MZ541784/ MZ541727/ MZ541668/ MZ541609. 17. ***P. chrysacanthus***. MX, Puebla: *DFE 33* (MEXU) MZ541785/ MZ541728/ MZ541669/ MZ541610. 18. ***P. chrysacanthus***. MX, Oaxaca: *DFE 34* (MEXU) MZ541786/ MZ541729/ MZ541670/ MZ541611. 19. ***P. chrysacanthus***. MX, Oaxaca: *DFE 35* (MEXU) MZ541787/ MZ541730/ MZ541671/ MZ541612. 20. ***P. chrysacanthus***. MX, Oaxaca: *DFE 36* (MEXU) MZ541788/ MZ541731/ MZ541672/ MZ541613. 21. ***P. chrysacanthus***. MX, Oaxaca: *DFE 37* (MEXU) MZ541789/ MZ541732/ MZ541673/ MZ541614. 22. ***P. chrysacanthus***. MX, Oaxaca: *DFE 38* (MEXU) MZ541790/ MZ541733/ MZ541674/ MZ541615. 23. ***P. chrysacanthus***. MX, Puebla: *DFE 42* (MEXU) MZ541791/ MZ541734/ MZ541675/ MZ541616. 24. ***P. chrysacanthus***. MX, Oaxaca: *DFE 64* (MEXU) MZ541792/ MZ541735/ MZ541676/ MZ541617. 25. ***P. collinsii*** (Britton & Rose) Byles & G.D. Rowley. MX, Oaxaca: *DFE 39* (MEXU) MZ541793/ MZ541736/ MZ541677/ MZ541618. 26. ***P. collinsii***. MX, Oaxaca: *DFE 40* (MEXU) MZ541794/ MZ541737/ MZ541678/ MZ541619. 27. ***P. collinsii***. MX, Oaxaca: *DFE 65* (MEXU) MZ541795/ MZ541738/ MZ541679/ MZ541620. 28. ***P. collinsii***. MX, Oaxaca: *DFE 65–2* (MEXU) MZ541796/ MZ541739/ MZ541680/ MZ541621. 29. ***P. collinsii***. MX, Oaxaca: *DFE 66* (MEXU) MZ541797/ MZ541740/ MZ541681/ MZ541622. 30. ***P. collinsii***. MX, Oaxaca: *DFE 67* (MEXU) MZ541798/ MZ541741/ MZ541682/ MZ541623. 31. ***P. collinsii***. MX, Oaxaca: *DFE 67–2* (MEXU) MZ541799/ MZ541742/ MZ541683/ MZ541624. 32. ***P. collinsii***. MX, Oaxaca: *DFE 68* (MEXU) MZ541800/ MZ541743/ MZ541684/ MZ541625. 33. ***P. collinsii***. MX, Oaxaca: *DFE 68–2* (MEXU) MZ541801/ MZ541744/ MZ541685/ MZ541626. 34. ***P. collinsii***. MX, Oaxaca: *DFE 69* (MEXU) MZ541802/ MZ541745/ MZ541686/ MZ541627. 35. ***P. gaumeri*** (Britton & Rose) Backeb. MX, Yucatán: *DFE 70* (MEXU) MZ541803/ MZ541746/ MZ541687/ MZ541628. 36. ***P. gaumeri***. MX, Yucatán: *DFE 71* (MEXU) MZ541804/ –/ MZ541688/ MZ541629. 37. ***P. gaumeri***. MX, Yucatán: *DFE 72* (MEXU) MZ541805/ MZ541747/ MZ541689/ MZ541630. 38. ***P. gaumeri***. MX, Yucatán: *DFE 73*

(MEXU) MZ541806/ MZ541748/ MZ541690/ MZ541631. 39. ***P. gaumeri***. MX, Yucatán: *DFE 73–2* (MEXU) MZ541807/ MZ541749/ MZ541691/ MZ541632. 40. ***P. gaumeri***. MX, Yucatán: *DFE 74* (MEXU) MZ541808/ MZ541750/ MZ541692/ MZ541633. 41. ***P. leucocephalus*** (Poselg.) Byles & G.D. Rowley. MX, Querétaro: *DA 281* (MEXU) MZ541809/ MZ541751/ MZ541693/ MZ541634. 42. ***P. leucocephalus***. MX, Chiapas: *DFE 41* (MEXU) MZ541810/ MZ541752/ MZ541694/ MZ541635. 43. ***P. leucocephalus***. MX, Veracruz: *DFE 43* (MEXU) MZ541811/ MZ541753/ MZ541695/ MZ541636. 44. ***P. leucocephalus***. MX, Veracruz: *DFE 44* (MEXU) MZ541812/ MZ541754/ MZ541696/ MZ541637. 45. ***P. leucocephalus***. MX, Veracruz: *DFE 45* (MEXU) MZ541813/ MZ541755/ MZ541697/ MZ541638. 46. ***P. leucocephalus***. MX, Veracruz: *DFE 46* (MEXU) MZ541814/ MZ541756/ MZ541698/ MZ541639. 47. ***P. leucocephalus***. MX, Querétaro: *DFE 47* (MEXU) MZ541815/ MZ541757/ MZ541699/ MZ541640. 48. ***P. leucocephalus***. MX, Querétaro: *DFE 47–2* (MEXU) MZ541816/ MZ541758/ MZ541700/ MZ541641. 49. ***P. leucocephalus***. MX, San Luis Potosí: *DFE 48* (MEXU) MZ541817/ MZ541759/ MZ541701/ MZ541642. 50. ***P. leucocephalus***. MX, San Luis Potosí: *DFE 49* (MEXU) MZ541818/ MZ541760/ MZ541702/ MZ541643. 51. ***P. leucocephalus***. MX, Tamaulipas: *DFE 58* (MEXU) MZ541819/ –/ MZ541703/ MZ541644. 52. ***P. leucocephalus***. MX, Tamaulipas: *DFE 59* (MEXU) MZ541820/ MZ541761/ MZ541704/ MZ541645. 53. ***P. leucocephalus***. MX, Tamaulipas: *DFE 60* (MEXU) MZ541821/ MZ541762/ MZ541705/ MZ541646. 54. ***P. leucocephalus***. SV, Chalatenango: *JM 2997* (MHES) MZ541822/ MZ541763/ MZ541706/ MZ541647. 55. ***P. leucocephalus***. SV, Santa Ana: *JM 3002* (MHES) MZ541823/ MZ541764/ MZ541707/ MZ541648. 56. ***P. leucocephalus***. HN, Olancho: *LF 736* (TEFH) MZ541824/ –/ MZ541708/ MZ541649. 57. ***P. leucocephalus***. HN, Comayagua: *LF 749* (TEFH) MZ541825/ MZ541765/ MZ541709/ MZ541650. 58. ***P. millspaughii*** (Britton) Byles & G.D. Rowley. CU, Las Tunas: *Barrios et al*. HFC88503 (HAJB) MZ541826/ –/ –/ –. 59. ***P. millspaughii***. CU, Holguín: *Barrios et al*. HFC88756 (HAJB) MZ541827/ MZ541766/ –/ –. 60. ***P. moritzianus*** (Otto) Byles & G.D. Rowley. VE, Aragua: *G. Carnevali* CR–2759, cult. MZ541828/ MZ541767/ MZ541710/ MZ541651. 61. ***P. pachycladus*** F. Ritter. BR, JQ889309/ –/ KX387753/ –. 62. ***P. purpusii*** (Britton & Rose) Byles & G.D. Rowley. MX, Colima: *DFE 50* (MEXU) MZ541829/ MZ541768/ MZ541711/ MZ541652. 63. ***P. purpusii***. MX, Colima: *DFE 50–2* (MEXU) MZ541830/ MZ541769/ MZ541712/ MZ541653. 64. ***P. purpusii***. MX, Jalisco: *DFE 51* (MEXU) MZ541831/ MZ541770/ MZ541713/ MZ541654. 65. ***P. purpusii***. MX, Jalisco: *DFE 53* (MEXU) MZ541832/ –/ MZ541714/ MZ541655. 66. ***P. purpusii***. MX, Jalisco: *DFE 54* (MEXU) MZ541833/ MZ541771/ MZ541715/ MZ541656. 67. ***P. purpusii***. MX, Sinaloa: *DFE 63* (MEXU) MZ541834/ MZ541772/ MZ541716/ MZ541657. 68. ***P. purpusii***. MX, Sinaloa: *DFE 63–2* (MEXU) MZ541835/ MZ541773/ MZ541717/ MZ541658. 69. ***P. purpusii***. MX, Jalisco: *DS 478–B* (IBUG) MZ541836/ MZ541774/ MZ541718/ MZ541659. 70. ***P. robinii*** (Lem.) Byles & G.D. Rowley. CU, Mayabeque: *Barrios et al*. HFC88800 (HAJB) MZ541837/ –/ –/ –. 71. ***P. vilaboensis*** (Diers & Esteves) P.J. Braun. BR, –/ JN035535/ KX387745/ –. 72. ***Rebutia minuscula*** K. Schum. AR, *B.O.Schlumpberger x108* (M) JQ779813/ JQ779677/ –/ –. 73. ***Stetsonia coryne*** (Salm-Dyck) Britton & Rose. AR, *B.O.Schlumpberger x57* (M) JQ779806/ JQ779670/ –/ –. 74. ***Xiquexique gounellei*** (F.A.C. Weber ex K. Schum.) Lavor & Calvente. BR, Bahía: CS–128, cult. MZ541838/ MZ541775/ KX301113/ MZ541660.

Appendix S2. List of morphological characters and states of character incorporate in the total evidence analysis for the *Pilosocereus leucocephalus* group *s.s.* To reference the branch, spines and fruit color we use the world color survey chart (http://www1.icsi.berkeley.edu/wcs/data.html, accessed 29 November 2019)

1. Branch diameter (Tt, $P < 0.001$): (0) > 6 cm, (1) ≤ 6 cm.
2. Areole length (Tt, $P < 0.001$): (0) > 3.5 mm, (1) ≤ 3.5 mm.
3. Areole width (Tt, $P < 0.001$): (0) > 3.5 mm, (1) ≤ 3.5 mm.
4. Areole length-width ratio (Tt, $P < 0.001$): (0) ≤ 1.3, (1) > 1.3.
5. Hairs: (0) absent, (1) present. The hairs are unbranched uniseriate trichomes, which are developed on flowering areoles in the apical portion of the branches and in some *Pilosocereus* species often constitute long dense tufts of hairs, e.g., in the *P. leucocephalus* group *s.s.*
6. Length of the longest hairs (Tt, $P < 0.001$): (0) ≤ 7 cm, (1) > 7 cm.
7. Habit: (0) shrubby, (1) treelike. Habit for family Cactaceae is the general aspect of a cactus, referred to treelike as a plant with lignified well-defined stem and shrubby as a plant with lignified basal branching stems.
8. Areole shape: (0) circular, (1) elliptic.
9. Branch color at the apex: (0) medium green (G15–17), (1) light green (D15–17), (2) light blue green (E18–20).
10. Spine color at the branch apex: (0) yellow (C8–9), (1) orange-brown (F5–7), (2) dark brown (I4–6).
11. Fertile part disposition: (0) discontinuous, (1) continuous. This character refers to separation evident between the long dense tufts of hairs. In some taxa evaluated, these long dense tufts of hairs usually not both overlapping those of adjacent areoles on a rib.
12. Fruit color: (0) purple (H36–38), (1) reddish (G1–3), (2) blue green (G21–24).
